# Meta-analysis identifies native priority as a mechanism that supports the restoration of invasion-resistant plant communities

**DOI:** 10.1038/s42003-023-05485-8

**Published:** 2023-10-30

**Authors:** Melinda Halassy, Péter Batáry, Anikó Csecserits, Katalin Török, Orsolya Valkó

**Affiliations:** 1 National Laboratory for Health Security, Centre for Ecological Research, Budapest, Hungary; 2 Institute of Ecology and Botany, Centre for Ecological Research, Vácrátót, Hungary; 3 ‘Lendület’ Landscape and Conservation Ecology Research Group, Institute of Ecology and Botany, Centre for Ecological Research, Vácrátót, Hungary; 4 ‘Lendület’ Seed Ecology Research Group, Institute of Ecology and Botany, Centre for Ecological Research, Vácrátót, Hungary

**Keywords:** Restoration ecology, Invasive species

## Abstract

The restoration of invasion-resistant plant communities is an important strategy to combat the negative impacts of alien invasions. Based on a systematic review and meta-analysis of seed-based ecological restoration experiments, here we demonstrate the potential of functional similarity, seeding density and priority effect in increasing invasion resistance. Our results indicate that native priority is the most promising mechanism to control invasion that can reduce the performance of invasive alien species by more than 50%. High-density seeding is effective in controlling invasive species, but threshold seeding rates may exist. Overall seeding functionally similar species do not have a significant effect. Generally, the impacts are more pronounced on perennial and grassy invaders and on the short-term. Our results suggest that biotic resistance can be best enhanced by the early introduction of native plant species during restoration. Seeding of a single species with high functional similarity to invasive alien species is unpromising, and instead, preference should be given to high-density multifunctional seed mixtures, possibly including native species favored by the priority effect. We highlight the need to integrate research across geographical regions, global invasive species and potential resistance mechanisms.

## Abstract (web summary)

A systematic review and meta-analyses of seed-based ecological restoration experiments identify native priority as a promising mechanism for controlling invasion of alien plant species, which can reduce the performance of invasive species by more than 50%.

## Introduction

Biological invasion is considered to be one of the main drivers of biodiversity loss with potential negative socio-economic impacts^1–3^. Ecological restoration is increasingly recognized as a relevant tool to combat land degradation and biodiversity loss^4^, and direct control of invasive alien species is often part of restoration projects^5^. However, restorative activities themselves involve disturbances such as soil disturbance or vegetation control leading to higher resource availability and reduced competition, and thus to increased invasibility^6^. Invasive alien plant species are well adapted to rapid establishment and exploitation of the post-disturbance environment, and primary or secondary invasion may therefore be a serious obstacle to the recovery or restoration of natural vegetation^7,8^. It has been suggested that the rapid establishment of a competitive native vegetation cover can reduce the invasion and spread of alien plants (see Hess et al. ^9^ and citations within) mainly through niche pre-emption and resource acquisition resulting in increased biotic resistance^10,11^. Biotic resistance is defined as the ability of a community to hinder the establishment of later arriving species, applied also to the establishment of invasive alien species^12^. The original term used in the invasion literature refers to the ability of existing native communities to oppose new, alien invaders^13^, and it refers to “the site biotic characteristics” of the invasion triangle, but not to the attributes of the invader species (including propagule pressure) or site environmental conditions^14^.

Understanding the mechanisms of biotic resistance may help design seed-based restoration methods to prevent and mitigate alien invasion. Several factors can be responsible for the competitive exclusion of invasive alien species^15^. One of the earliest phenomena discussed in the literature is the diversity-resistance effect^12^, which implies that more diverse communities utilize resources more completely, limiting the resources for later invaders^16,17^. Functional similarity may further increase the competitive exclusion of invasive alien species by native species^18,19^. The original theory of limiting similarity^20^ explains how coexistence is possible in multi-species communities through the differences in the use of resources by species (niche differentiation). Consequently, species that use the same resources similarly cannot coexist stably (competitive exclusion). Thus, theoretically, the integration of native species that functionally more closely resemble known high-risk invasive alien species into the restored communities may result in better resistance to invasion^21–23^. There is limited knowledge on how exactly the limiting similarity hypothesis should be applied to increase biotic resistance^24^ and what specific traits to be involved in such a design^11^. A meta-analysis by Price and Pärtel^25^ showed that most studies used very broad functional categories that do not necessarily reflect the traits important for competitive exclusion, such as the traits resulting in similar resource acquisition strategies. Laughlin^23^ proposed considering multiple functional traits simultaneously, however, this approach was not proved satisfactory in experimental set-up compared to the increased seed density of native species^26^.

Propagule pressure, including the abundance and frequency of arrival of individuals of an invasive alien species at a given time and location, was identified as a primary determinant of species invasiveness and habitat invasibility^27,28^ and was suggested as a null model for studies of biological invasions^29^. A higher number of introductions or a higher immigration rate increases the chances of establishment, niche occupation and resource acquisition^30–32^. Based on this phenomenon, density-driven suppression of invasive alien species is possible by increasing the seeding rates of native species to match the propagule pressure of invasive alien species. This, in turn, increases the chances of native establishment and the formation of a dense native cover resulting in better resource utilization in the resident community^33–36^.

More recently, the time factor of niche pre-emption has come into focus in attempts to restore invasion-resistant native communities. Namely, differences in the time of arrival of different species can have a profound effect on their environment, and on the establishment, survival, growth or reproduction of later-arriving species and yet on community dynamics^37,38^. This phenomenon is referred to as priority effect^39^. Priority in the arrival order has an advantage also in early resource pre-emption that can strongly influence competition and survival^40,41^. Early arrival and resource use are often linked to better dispersal capacities, earlier germination and faster growth, and these properties were found to promote the success of invasive alien species over native ones^42^. This also implies that manipulating priority by introducing native species before the emergence of invasive alien species, e.g., through assisted dispersal, can be used to design communities that are more resistant to invasion^21,43–45^.

As this brief introduction shows, there is a wealth of literature on the theoretical background and empirical field observations of the different mechanisms related to biotic resistance. Part of the suggested mechanisms have been already reviewed, like diversity and invasibility^10,46,47^, functional diversity^15^, plant traits and invasion resistance^19,24,25^, or priority effects^43,44^. However, these are mostly qualitative reviews and do not strictly focus on the invasion of alien species, and most studies investigate what makes an invasive alien species successful rather than how to increase the biotic resistance of native communities for restoration purposes. The complexity of the native-invasive relationships also makes it very difficult to isolate the different mechanisms that shape biotic resistance based on observations, so we have focused our systematic review on experimental approaches. We build on the work of previous reviews and add recent information, especially focusing on invasive alien species and their possible suppression by seeding native species in ecological restoration.

We performed a meta-analysis in the frame of a systematic review to provide an overview of current trends and future prospects for increasing biological resistance to invasive alien species in ecological restoration based on functional similarity, seeding density and native species priority. First, we tested the overall summary effect of seed-based ecological restoration on functional similarity, seeding density, and native species priority in controlling the establishment and growth of invasive alien species. Second, we tested the effect of increased functional similarity, seeding density and priority effect separately in three separate models. We set up the following hypotheses: 1. Based on the limiting similarity hypothesis, seeding of native species with higher functional similarity to invasive alien species will increase biotic resistance. 2. The higher the seeding density of native species, the more likely to form dense cover, resulting in quicker resource pre-emption that creates higher biotic resistance. 3. Since early-arriving species can strongly influence the performance of those arriving later in a system, establishing native species before the emergence of invasive alien species will increase biotic resistance. We also investigated the effects of moderators, namely the impact of focal species and experimental design on the effectiveness of restoration, as the invasion success can vary depending on the lifespan and growth form of the invader, as well as the time^48^ and experimental setup^49^. Finally, we discuss how functional similarity, seeding density, and priority effect can be applied in seed-based restoration practices to prevent and mitigate the spread of invasive alien species and future research perspectives.

## Results

### Characteristics of the reviewed studies

**Fig. 1:**
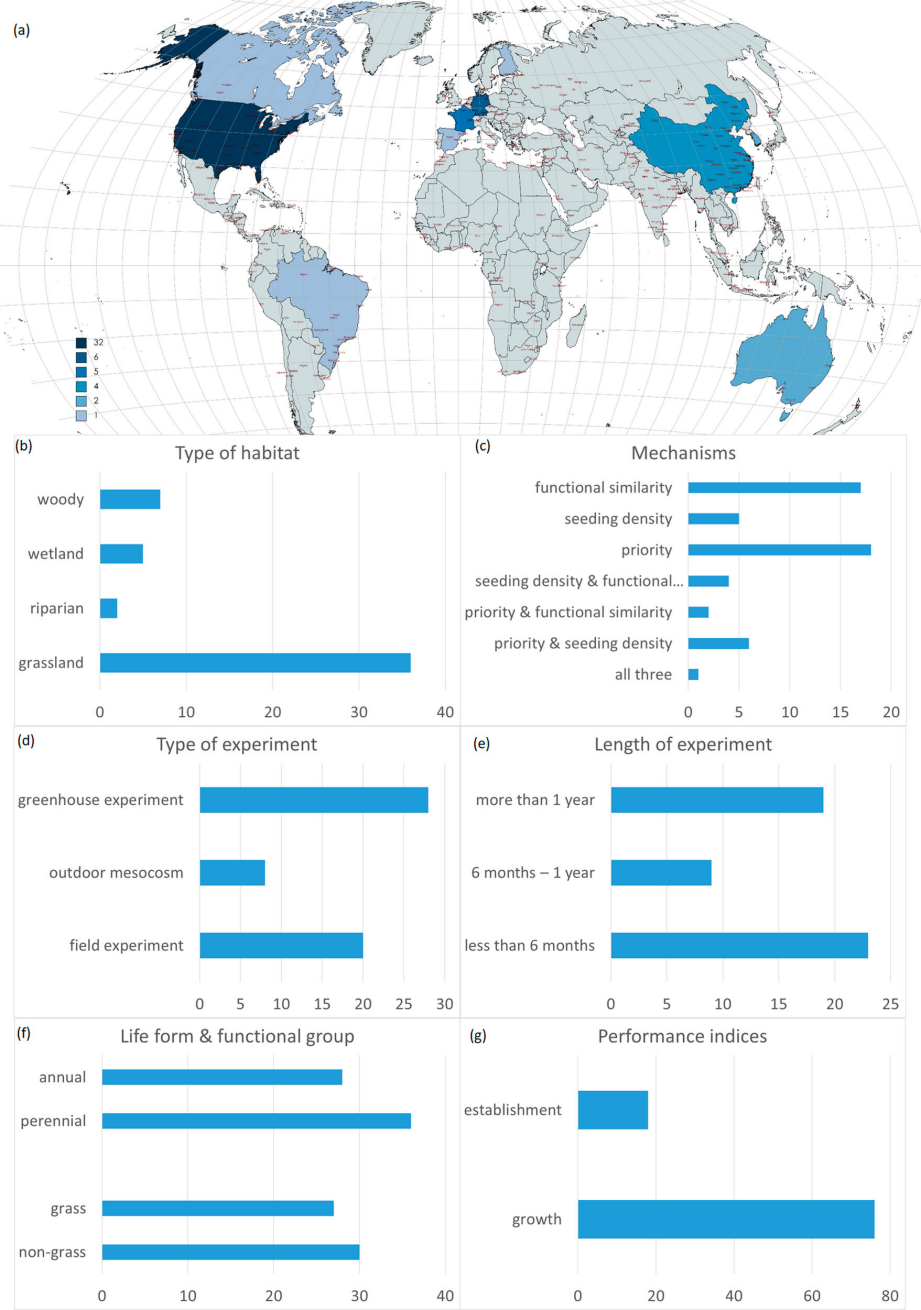
Geographical distribution and experimental design of the reviewed studies. The number of studies **a** per country, **b** habitat type, **c** studied mechanism, **d** experiment type, **e** experiment length, **f** life form and functional group of invasive alien species, and **g** performance indicators. The map was created using Winkel Tripel projection at https://www.mapchart.net. Source data can be found in Supplementary Data 2.

We identified 48 papers published between 1997 and 2022 based on our systematic review (Supplementary Table 1, Supplementary Figure 1). The data is strongly biased geographically towards the Northern Hemisphere (Fig. 1), with 5, 12 and 33 articles located in Asia, Europe, and North America, respectively, and only 3 papers from the Southern Hemisphere (South America: 1, Australia: 2). The habitats involved are mostly grassland habitats (36), including some riparian (2) or wetland habitats (5), plus woody steppe or woodland (7). The studied invasive alien species are very diverse regarding their life forms, functional groups, and, opposite to our expectations, more studies involved perennial species (31 species) than annual ones (24 species), and ca. half of the papers used multiple species. 18 papers included field experiments, 22 were greenhouse experiments (including heated, unheated and mesh-walled greenhouses) and 9 outdoor mesocosm experiments, and 2 papers included more types with comparative results. The length of the experiments varied between 30 days^50^ and 7 years^51^. Most papers studied the impact of native species on invasive abundance (e.g., biomass or cover: 76 data points), and fewer studied establishment (18). 14 papers focused on priority effect, only 4 on seeding density, 14 on functional similarity, 13 papers included the impact of two mechanisms together, and 1 paper encompassed all three mechanisms within one experiment.

### Overall summary effect of functional similarity, seeding density, and native species priority

**Fig. 2:**
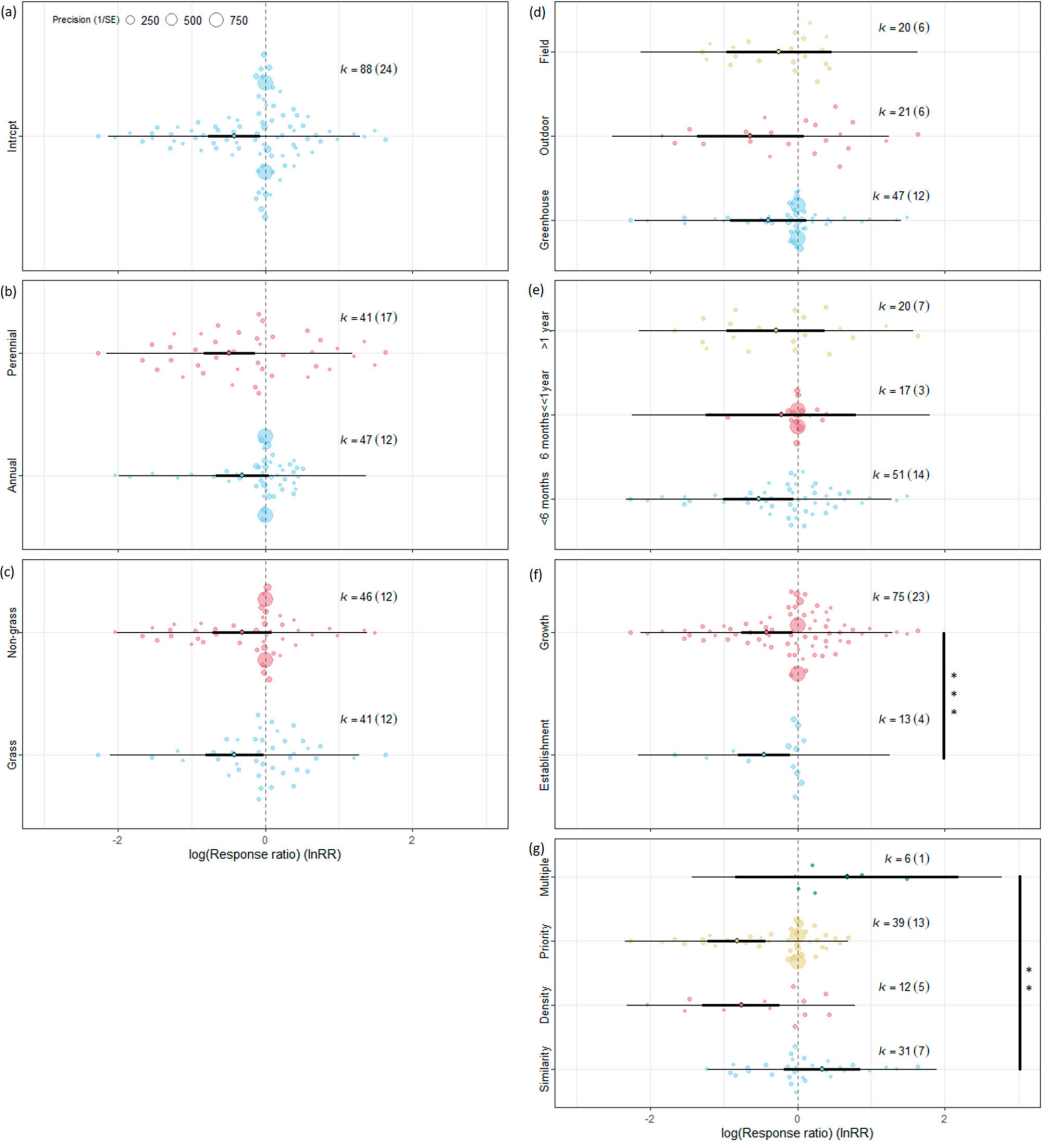
Orchard plot of the focal model of the suppression of invasive alien species (IAS) by seeding native species. Focal model includes all three mechanisms with IAS seeded alone as control. **a** Focal model without moderators. Moderators: **b** IAS life form (annual/perennial), **c** IAS functional type (grass/non-grass), **d** experiment type (field, outdoor, greenhouse), **e** experiment length (less than 6 months, between 6 months and 1 year, more than 1 year), **f** indicator type (establishment/growth), and **g** studied mechanisms (similarity, density, priority, multiple). Vertical dashed line means that the performance of IAS is the same with or without seeding native species. The line in bold represents the 95% confidence interval, the thin line represents the 95% prediction interval. If the confidence bar falls in the negative side and does not intersect with zero, we interpret that seeding native species affects IAS negatively. k=number of effect sizes used to derive the displayed statistics. Numbers in parentheses following k indicate the number of studies included in calculations. Significant between-group heterogeneity is signed. Signif. codes: 0 ‘***’, 0.001 ‘**’, 0.01 ‘*’ 0.05 ‘.’. Source data can be found in Supplementary Data 2.

We included 88 separate effect sizes calculated from 24 publications with available data in the focal model, where we used invasive alien species seeded alone as control (Supplementary Data 1). In the focal model (df = 87, Q = 1091.15, *p* < 0.001), a significant negative impact (*p* < 0.01) of seeding density and native priority was found that can reduce invasive performance by 50% (Table 1, Fig. 2, Supplementary Data 2, Supplementary Table 2). A significant negative impact (*p* < 0.05) was also confirmed for perennial and grass species, short-term studies and regarding the establishment of invasive alien species (Supplementary Table 2). From the studied moderators, we found a significant difference between the studied mechanisms (df = 3, Q = 16.56, *p* < 0.001). Manipulation of the seeding density and providing native priority was significantly (*p* < 0.05) more effective than functional similarity and multiple mechanisms (including functional similarity) (Supplementary Table 3).

**Table 1:** Summary table of meta-analysis models showing total heterogeneity, plus heterogeneities explained by moderators with corresponding residual heterogeneities for the focal model and models focusing on functional similarity, seeding density or priority effect.

*Moderator*		*Focal study*		*Functional similarity*		*Seeding density*		*Priority effect*	
		*Moderator*	*Residuals*	*Moderator*	*Residuals*	*Moderator*	*Residuals*	*Moderator*	*Residuals*
*None*	*df*	87		37		18		28	
	*Q*	1091.15		251.9482		563.1752		703.8589	
	*p*	<0.001		<0.001		<0.001		<0.001	
*Mechanism*	*df*	**3**	84						
	*Q*	**16.56**	1046.73						
	*p*	**0.001**	<0.001						
*IAS life form*	*df*	1	86	1	36	1	17	**1**	27
	*Q*	3.73	617.67	0.69	190.36	1.57	454.51	**4.54**	683.83
	*p*	0.053	<0.001	0.406	<0.001	0.211	<0.001	**0.033**	<0.001
*IAS functional type*	*df*	1	85	1	36	1	17	1	26
	*Q*	0.30	1071.11	0.16	250.52	0.94	339.30	0.84	554.09
	*p*	0.586	<0.001	0.693	<0.001	0.333	<0.001	0.360	<0.001
*Experiment type*	*df*	2	85	2	35	**2**	16	2	26
	*Q*	0.57	945.68	0.64	237.50	**21.60**	109.06	0.27	552.40
	*p*	0.751	<0.001	0.728	<0.001	< **0.001**	<0.001	0.876	<0.001
*Experiment length*	*df*	2	85	2	35	2	16	2	26
	*Q*	0.49	907.89	2.86	155.25	1.61	334.54	5.77	461.63
	*p*	0.784	<0.001	0.239	<0.001	0.447	<0.001	0.056	<0.001
*IAS performance indicator*	*df*	**1**	86	**1**	36	1	17	1	27
	*Q*	**18.64**	1073.68	**8.39**	247.65	0.09	490.32	3.52	702.75
	*p*	< **0.001**	<0.001	**0.004**	<0.001	0.765	<0.001	0.061	<0.001

‘None’ refers to the performance of invasive alien species (IAS) sown together with native species compared to invasive alien species seeded alone without any moderator (focal model). Moderators: Mechanism (functional similarity, seeding density, priority effect, and multiple), IAS life form (annual/perennial) and functional type (grass/non-grass), Experiment type (field, outdoor, greenhouse) and length (less than 6 months, between 6 months and 1 year, more than 1 year), and IAS performance indicator type used (establishment/growth). Significant (p < 0.05) moderator effects are shown in bold.

### Functional similarity model

**Fig. 3:**
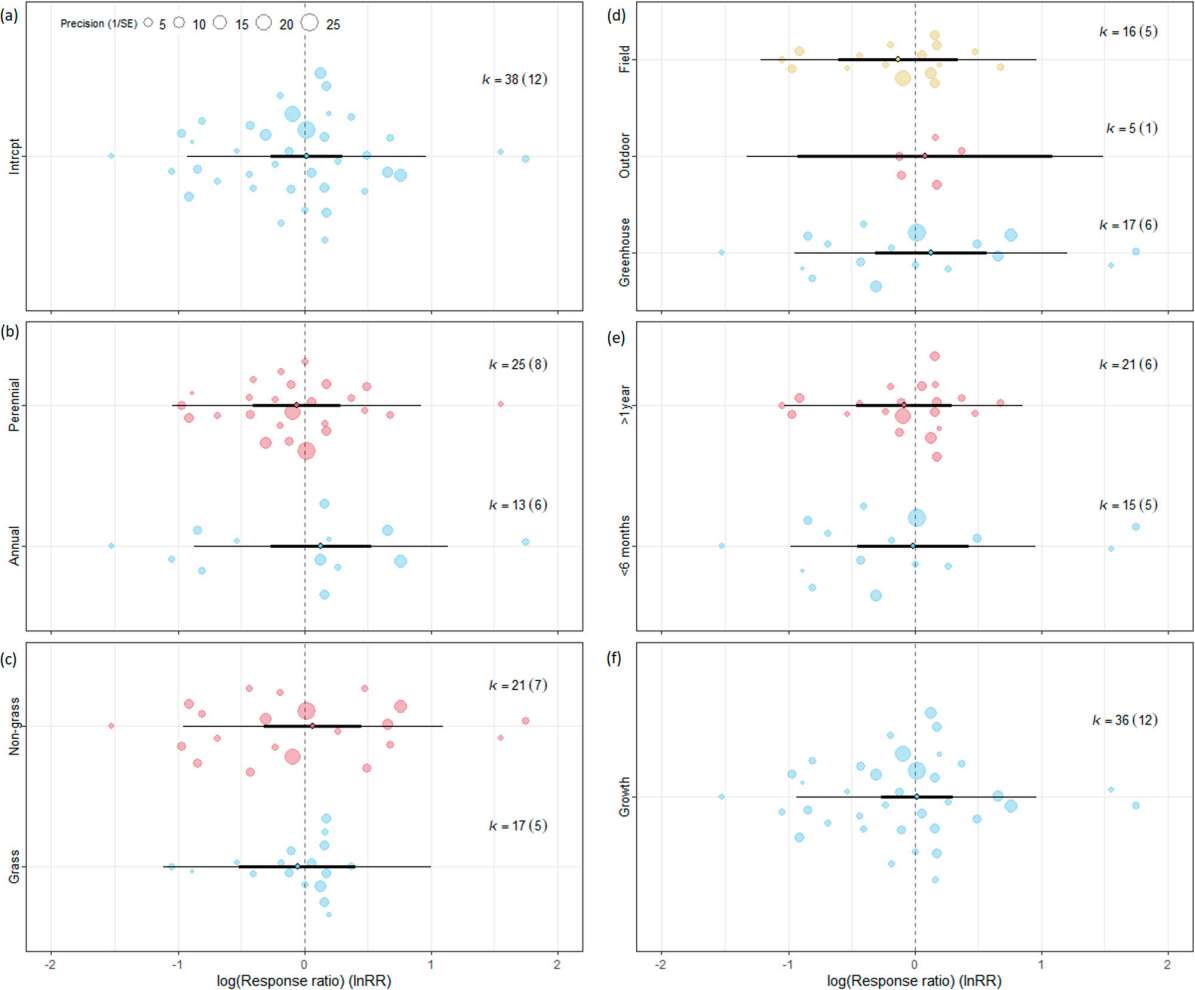
Orchard plot of functional similarity model of the suppression of invasive alien species (IAS) by seeding native species. Functional similarity model includes seeding low similarity native species with IAS as control. **a** Focal model without moderators. Moderators: **b** IAS life form (annual/perennial), **c** IAS functional type (grass/non-grass), **d** experiment type (field, outdoor, greenhouse), **e** experiment length (less than 6 months, between 6 months and 1 year, more than 1 year), **f** indicator type (establishment/growth). Vertical dashed line means that the performance of IAS is the same with or without seeding native species. The line in bold represents the 95% confidence interval, the thin line represents the 95% prediction interval. If the confidence bar falls in the negative side and does not intersect with zero, we interpret that seeding native species affects IAS negatively. k=number of effect sizes used to derive the displayed statistics. Numbers in parentheses following k indicate the number of studies included in calculations. Significant between-group heterogeneity is signed. Signif. codes: 0 ‘***’, 0.001 ‘**’, 0.01 ‘*’ 0.05 ‘.’. Please note data series with k < 3 (establishment and experiments between 6 months and 1 year) were removed. Source data can be found in Supplementary Data 2.

Based on 12 publications with 38 data points, high functional similarity compared to low functional similarity did not affect negatively invasive alien species (Table 1, Fig. 3, Supplementary Data 2, Supplementary Table 4). At the case study level, the majority of studies found partial or no support for the limiting similarity hypothesis (Supplementary Data 1). From the moderators, only the type of indicator used had a significant impact (df = 1, Q = 8.39, p = 0.004). Seeding functionally similar native species was highly beneficial for the establishment of invasive alien species (please note that the number of data points was very low) and neutral for their growth.

### Seeding density model

**Fig. 4:**
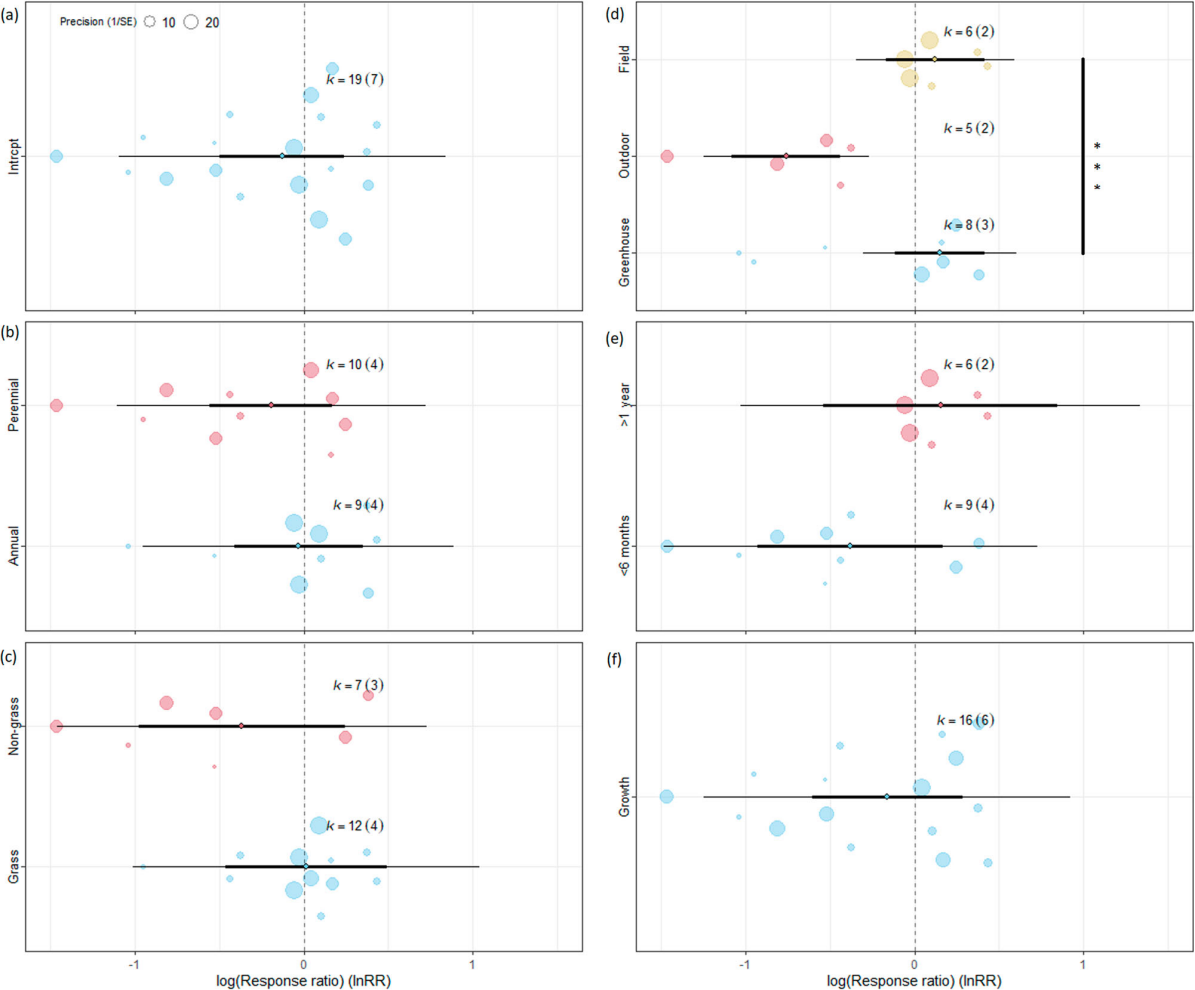
Orchard plot of seeding density model of the suppression of invasive alien species (IAS) by seeding native species. Seeding density model includes low-density seeding of native species with IAS as control. **a** Focal model without moderators. Moderators: **b** IAS life form (annual/perennial), **c** IAS functional type (grass/non-grass), **d** experiment type (field, outdoor, greenhouse), **e** experiment length (less than 6 months, between 6 months and 1 year, more than 1 year), **f** indicator type (establishment/growth). Vertical dashed line means that the performance of IAS is the same with or without seeding native species. The line in bold represents the 95% confidence interval, the thin line represents the 95% prediction interval. If the confidence bar falls in the negative side and does not intersect with zero, we interpret that seeding native species affects IAS negatively. k=number of effect sizes used to derive the displayed statistics. Numbers in parentheses following k indicate the number of studies included in calculations. Significant between-group heterogeneity is signed. Signif. codes: 0 ‘***’, 0.001 ‘**’, 0.01 ‘*’ 0.05 ‘.’. Please note data series with k < 3 (establishment and experiments between 6 months and 1 year) were removed. Source data can be found in Supplementary Data 2.

Although many studies report the beneficial impact of high-density seeding on increasing biotic resistance in general^27,28^, we found only 7 papers with 19 data points on the impact of increased native seed densities on experimentally introduced invasive alien species (Supplementary Data 1). Based on this dataset, increasing the seeding density of native species did not result in increased suppression of invasive alien species (Table 1, Fig. 4, Supplementary Data 2, Supplementary Table 5). The density effect was only confirmed for outdoor mesocosm (*p* < 0.0001) where the performance of invasive alien species was reduced by half (significantly different from greenhouse and field experiments at *p* < 0.01) (Supplementary Table 6).

### Priority effect model

**Fig. 5:**
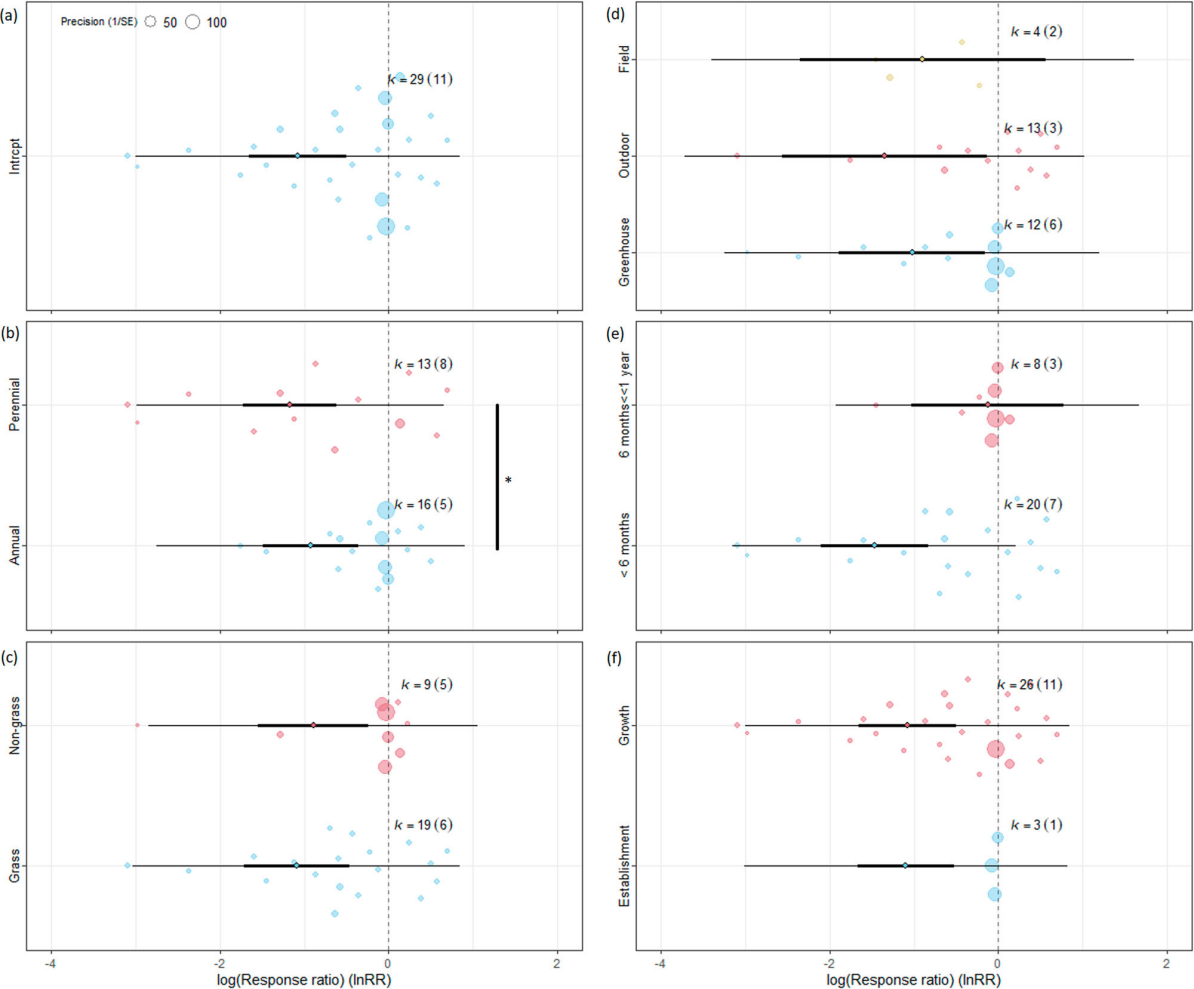
Orchard plot of priority effect model of the suppression of invasive alien species (IAS) by seeding native species. Effect model includes low-priority seeding of native species with IAS as control. **a** Focal model without moderators. Moderators: **b** IAS life form (annual/perennial), **c** IAS functional type (grass/non-grass), **d** experiment type (field, outdoor, greenhouse), **e** experiment length (less than 6 months, between 6 months and 1 year, more than 1 year), **f** indicator type (establishment/growth). Vertical dashed line means that the performance of IAS is the same with or without seeding native species. The line in bold represents the 95% confidence interval, and the thin line represents the 95% prediction interval. If the confidence bar falls on the negative side and does not intersect with zero, we interpret that seeding native species affects IAS negatively. k=number of effect sizes used to derive the displayed statistics. Numbers in parentheses following k indicate the number of studies included in calculations. Significant between-group heterogeneity is signed. Signif. codes: 0 ‘***’, 0.001 ‘**’, 0.01 ‘*’ 0.05 ‘.’. Please note data series with k < 3 (experiments lasting more than 1 year) were removed. Source data can be found in Supplementary Data 2.

Our meta-analysis based on 11 publications with 29 data points confirmed that giving native species a priority can significantly (*p* = 0.0002) suppress invasive alien species, by more than 60% (Table 1, Fig. 5, Supplementary Data 1 and 2). A significant negative impact (*p* < 0.05) was confirmed for all moderator variables, expect for field experiments and longer-term experiments (more than 6 months) (Supplementary Table 7). We found no significant impact of moderators except for life forms (df = 1, Q = 4.54, *p* = 0.33) where perennial species were affected slightly more negatively (60% and 70% reduction, respectively).

## Discussion

Based on our quantitative review of 48 papers published in relation to seed-based ecological restoration experiments, we demonstrate the potential of seed-based ecological restoration in controlling the establishment and growth of invasive alien species up to 40%. From our three hypotheses, we had to reject the hypothesis that the introduction of native species with higher functional similarity to invasive alien species increases biotic resistance. Seeding functionally similar species generally had a neutral effect and was even highly beneficial for the establishment of invasive alien species. Our second hypothesis regarding increased seeding density was partly confirmed. High-density seeding is effective in reducing invasive species compared to the control, but this effect was not significant compared to lower seeding densities. We confirmed our third hypothesis that native priority increases biotic resistance. Giving priority to native species reduced the performance of invasive alien species the most, by more than 50% both compared to control and to low-priority seeding. Generally, the impacts were more pronounced on perennial and grassy invaders and on the short-term.

Based on our study, giving priority to native species was found to be the best approach in increasing the biotic resistance of native communities to invasion, whereas increasing functional similarity or seeding density did not increase invasion resistance. Most studies on the priority effect, including native and invasive alien species, confirm the benefits of arriving early and the cost of arriving late, thus ensuring the priority of native species can help decrease invasion. Even a short-term advantage (even as little as one week) can strongly influence the outcome of competition between native species and invasive alien species, but the priority effect can be strengthened by increasing the time advantage given to native species^43,44^. At the same time arriving early often benefits invasive alien species more and costs them less than native species^43,44^. The early advantage of invasive alien species can be explained by their high germination success, earlier emergence, higher germination and growth rates, and greater light capture during the early stages of establishment compared to native species^42^. Furthermore, invasive alien species more often create soil legacies than native species that hamper later arriving species^43^. Tolerance of invasive alien species to late arrival can be explained by a better tolerance to resource limitation^52^, or higher niche breadth or better competitive abilities than native species^13,19,43,44^.

Manipulating the composition of native species can strongly influence community invasibility, but according to our meta-analyses, and similar to previous reviews^19,24,25^, functional similarity between native and invasive alien species does not strengthen this impact. There are several reasons why experiments fail to demonstrate the impact of limiting similarity. Often, in an experimental setup, resources are not limited^49^, so there is no strong competition. Traits should be selected according to their potential impact on community assembly, which depends both on the studied habitat and the focal invasive alien species^19^. Selecting species based on functional similarity is challenging because invasive alien species are often found at the edges of the trait range occupied by native species^11^ and have combinations of traits that native plants do not^53^. This also implies that instead of a single-trait and single-species approach, multifunctional seed mixtures covering a wide spectrum of traits should be prioritized possibly including native species advantaged by the priority effect^19^. Finally, achieving a high degree of native-invasive functional similarity is difficult, and prioritizing functional similarity can often only be achieved at the expense of community biomass, while not increasing biotic resistance if resources are abundant^54^. Further research is needed to determine how to optimize species composition and if functional similarity can be better applied in the practice of ecological restoration.

When resources are not limited, as is often the case in ecological restoration, where habitats are disturbed and disturbance is usually associated with increased nutrient availability, density-driven suppression of invasive alien species can be an alternative solution^33,55^. Increasing the seeding density of native species generally leads to increased seedling establishment and biomass of native species that, in turn, increase invasion resistance^33,54,55^. However, we found that density-driven suppression of invasive alien species is less relevant than native priority. A possible explanation is that seedling and especially adult density does not have a linear relationship with increasing seeding density, rather threshold densities could exist^56,57^, above which increased seeding densities cannot result in increased seedling density due to the limited availability of safe sites^58^ or density-dependent mortality^59^.

The variability found in the applicability of functional similarity, seeding density, and priority effect in increasing biotic resistance can be in part due to the differences in experimental arrangements^48,49^. Considering the invasive alien species, we found a negative impact only on perennial and grass species in our focal study, but the impacts on different life forms and functional groups were not significantly different. Grasses (among them the genus *Bromus*) are the most frequently controlled invasive alien species worldwide^5^ and our results suggest that it is possible to increase biotic resistance to them. Regarding the experimental settings, although negative impacts were found only under outdoor (seeding density and priority effect) and greenhouse conditions (priority effect) and in the short term (focal study, priority effect), results did not depend significantly on the type and length of experiments. A significant difference was found depending on performance indicators in the focal study and in the functional similarity study, but the results are contradictory. We have found weak evidence that the establishment of invasive alien species may even be facilitated by the presence of similar native species, if only functional similarity is considered. This is in line with earlier findings that the introduction of native species cannot completely outcompete invasive species, but it can only reduce their long-term performance^10^. However, the establishment of invasive species was negatively affected when considering all mechanisms together.

In practice, the success of invasion is determined by the interaction of invasive propagule pressure, biotic resistance, and environmental conditions^14^. The first step in an integrated approach to invasion would be prevention^7^. In ecological restoration, sites are inevitably disturbed either by factors that lead to degradation or restoration activities themselves, resulting in greater resource availability and less competition. Native plant communities at an early stage of development are not able to take full advantage of available resources, leaving room for invasive alien species^8^. Studies on priority effect show that early arrival greatly benefits invasive alien species, which can even cause legacies in the system^43,44^. Minimizing disturbance is the first step to prevent the spread of invasive alien species, followed by reducing their propagule pressure. The latter can be done either by prioritizing restoration in areas with low invasion levels^60^ or by actively controlling propagule pressure directly (by removing the soil seed bank and vegetative parts) or indirectly (by early weed control, which reduces the number of produced seeds and can create a temporal priority for native species)^43,44^.

Biotic resistance can be best increased by the early introduction of early-emerging, fast-growing native species, and high-yielding communities that are suggested to achieve early niche pre-emption and lower the risk of invasion^11,15,54^. Furthermore, the order of seeding of native species can also influence the restoration success. It is recommended to use grass species but avoid the early use of legume species, as these N_2_-fixing species can promote the establishment of invasive alien plants^9^. Sustainability of priority impacts can be achieved by hampering the establishment of invasive alien species, e.g., by including native species that can induce niche modification in addition to niche pre-emption^41^, such as species with known allelopathic effects^54^. Density-driven suppression of invasive alien species can be an alternative or complementary solution^55^. Raising seeding rates is more likely to create competitive native cover. However, seeding single fast-growing dominant species at high densities can also reduce native diversity^61^, therefore, high-density, functionally diverse seed mixes are better suited for restoration purposes^15^. Also, threshold constraints should be considered^56,57^, and a preliminary assessment may be needed before setting seeding rates. Further research is needed to determine whether a combination of different seeding strategies (including functional similarity) can help to strengthen resistance to invasion in both the short and longer term.

Limiting plant invasions offers further opportunities to reduce harmful insect invasions^62^. Since many invasive alien insect herbivores feed on invasive alien plants, a reduction in the number of host plants will also lead to a reduction in the number of invasive insect species. However, seeding of a single plant species in high densities can increase the risk of outbreak of the insects that it hosts. Instead, biotic resistance to invasive insect species may be enhanced by increasing native diversity, as a negative correlation has been found between host richness and invasion success at smaller spatial scales^19^.

It is important to note that even combining the best methods to increase biotic resistance in restoration would not result in the complete elimination of invasive alien species, but would limit their biomass and seed production, reducing the risk of further invasion^10^. To achieve sustainability, follow-up monitoring, resource manipulation, and selective control of invasive alien species is necessary^51^. The long-term success of restoration depends on community assembly processes that can be influenced by tackling all three sides of the invasion triangle^14^ by targeted removal of invasive alien species, increasing biotic resistance, and manipulating environmental conditions.

Here, we reviewed how biotic resistance can be enhanced through functional similarity, seeding density, and priority effect, however, due to limited data availability, we had no opportunity to address the role of other mechanisms responsible for biotic resistance, such as diversity, functional diversity, environmental factors or plant-animal relationships. The use of biotic resistance in restoration to prevent invasion is a new research direction, and our knowledge is limited compared to the invasiveness or invisibility of habitats^13,46,47^. Consequently, the focal species, resistance-enhancing mechanisms (including the levels of functional similarity, seeding density, and priority effect), and experimental design involved show a large variability without many replications, and therefore it is challenging to generalize the results. We also faced the problem of having limited access to data, as non-significant data is not published that has seriously hampered e.g. comparisons between multiple mechanisms. We found a significant bias with sample size for the functional similarity and seeding density studies (Supplementary Table 8) and a strong geographic bias with the Southern Hemisphere underrepresented and North America overrepresented which weakens the strength of our findings. Moreover, the studies conducted so far did not address those plant species or genera that are considered the most problematic globally^63^, except for one species (*Mikania micrantha*).

In order to increase the reliability of research on invasion resistance, we suggest the following considerations. Research collaborations should be established, working with the same species in its original range and in the new, invaded geographical region or the same species invading different regions. Further coordinated and more standardized research is needed to exploit the potential of invasion-resistant restoration better, e.g., standardization of functional similarity calculations, applied seeding rates, and priority levels. In addition to studying multiple species, multiple mechanisms need to be tested under similar experimental conditions to obtain comparable results for the various mechanisms of biotic resistance^9^.

Finally, incorporating and integrating the latest concepts and hypotheses on invasion^64^ is essential to improve the predictive capacity of invasion ecology and to identify best restoration practices to prevent and control invasive alien species.

## Methods

### Literature survey

We systematically screened the literature for restoration experimental studies on the biotic resistance of native species or communities towards invasive alien species due to functional similarity, seeding density, and native priority. We further refer to these as basic mechanisms responsible for biotic resistance. The search was performed using the ISI Web of Science database (Science Citation Index Expanded edition) on 16 March 2022 and then updated on 7 February 2023. We used the exact search feature with the search strings for invasion, active introduction of species and the three studied mechanisms of resistance: ALL = ((invasi*) AND (seeding OR sow* OR planting) AND (“functional similarity” OR “plant trait” OR “seed density” OR “seeding rate” OR “propagule pressure” OR “priority” OR “arrival order”)). We retained only publications in English with no limit for publication date. The search yielded 202 records.

We only retained articles that met the following criteria (see also our PICO model in Supplementary Table 1): (1) focused on restoration-oriented experimental studies conducted in terrestrial plant communities; (2) included active introduction of native species and invasive alien species via seeding or planting; (3) involved at least one native species and one invasive alien species; (4) and at least one of the following aspects:

- functional similarity: there were at least two different native species involved, which differ in functional similarity to the invasive alien species;
- seeding density: native species were sown at a minimum of two different seed densities;
- priority effect: native species were sown on at least two occasions, at the same time as and before invasive alien species.

The initial title screening that focused on experimental studies in terrestrial vegetation only reduced the list further down to 121. These items were then checked for the abstract, leaving 54 records where the full text was downloaded for a thorough analysis to determine whether they provided any answers to our research questions. This reading resulted in 31 papers. We additionally searched for relevant references in recent reviews on the topic^43,44^ and in the selected papers in our research. The search yielded additional 17 records. The PRISMA flow chart shows the whole screening process (Supplementary Figure 1).

### Data extraction

Mean, replication (N), and standard deviation estimates (sd, sem or 95% CI) for both control and treatment were compiled for the performance of invasive alien species. Data were extracted from text or tables or read from figures using the metaDigitise R package^65^. Data from the same publication, but for different species or mechanisms were collected as separate data points. For multi-year studies, only data from the last year were extracted. If multiple indicators were included in a publication, biomass was preferred for growth and seedling survival for establishment. The logarithmic response rate (lnRR) was calculated as an estimate of the effect size, as it is not affected by different variances between control and treated groups, and the results are easy to interpret^66^. The replication number was not available for one publication, which was excluded from our analysis. In some papers, relative competition intensity (RCI = (Pcontr − Ptreat)/Ptreat) was reported that we converted to lnRR using the following equitation: lnRR = ln(Pcontr/Ptreat) = −ln(1 − RCI)^67^.

Additionally, from each selected publication, we collected the following information: the publication year, the country, the study type (field or greenhouse experiment), the native species and invasive alien species involved, and the mechanisms studied. Special focus was on the details of the studied three basic mechanisms related to biotic resistance, such as the functional groups or plant traits considered, seeding densities used for native species and invasive alien species, and the difference between seeding/planting times of introduced plants used in studies on priority effect. We also collected information on the types of habitats involved, the plant form and number of individuals applied, the temporal and spatial scale of the experiments, the details of treatments and maintenance, and the performance indicators used.

### Statistics and reproducibility

To examine the heterogeneity of effect sizes, we performed a meta-analysis using mixed-effects models with moderators as predictor variables. The focal model included all three mechanisms together, where seeding of invasive alien species without native species was considered as control and high similarity, seeding density, and priority of native species as treatment. Additionally, we performed three separate models for each of the three mechanisms, using low similarity, low density, or simultaneous seeding as controls for functional similarity, seeding density, and priority effect, respectively. Moderators included the mechanisms (functional similarity, seeding density, priority effect or combined) studied for the focal analysis, plus life form (annual or perennial) and functional group (grass or non-grass) of the invader species, the type (field, outdoor or greenhouse) and length (less than 6 months, between 6 months and 1 year, more than 1 year) of the experiments and the main indicators (establishment or growth) of invasive performance. Study ID was treated as a random effect in all models to account for the non-independence of individual effect sizes calculated from the same study. Multiple comparisons between different moderator levels of mixed-effects models were performed using the generalized linear hypotheses (glht) function. We carried out sensitivity analyses for each model by checking results with and without including outliers defined using graphical methods (boxplots), and removed the outliers and data points with extremely high variability of lnRR. We compared models with and without outliers based on Akaike’s information criterion. The final number of papers and data points included in the models are: full model *n* = 24 publications and k = 88 data points, functional similarity model *n* = 12, k = 38, seeding density model *n* = 7, k = 19, and priority effect model *n* = 11, k = 29. R code examsamples and model results with or without outliers are presented in Supplementary Notes 1 and 2.

We assessed whether publication bias could be detected for the data included in the meta-analysis using funnel plots, Kendall’s rank correlation tests, and Egger’s tests. The funnel plots showing the effect size versus the standard error of the mean were visibly not highly skewed, and this was also confirmed by Kendall’s rank correlation statistics (Supplementary Figure 2). We found significant bias with sample size as a predictor based on Egger’s test when analyzing functional similarity and seeding density separately even after removing outliers (Supplementary Table 8).

All statistical analyses and graphical presentations were performed in the R programming environment Version 3.4.4^68^. by using R-packages metafor^69^, multicomp^70^ and orchaRd 2.0 package^71^.

### Reporting summary

Further information on research design is available in the Nature Portfolio Reporting Summary linked to this article.

### Supplementary information

Supplementary information

Description of Additional Supplementary Files

Supplementary Data 1

Supplementary Data 2

Reporting Summary

## Data Availability

The authors declare that the source data for the main Figures can be found in Supplementary Data 1 and 2. Any other relevant data are available from the corresponding author upon reasonable request.
