# Supplementary material for: Meta-analysis identifies native priority as a mechanism that supports the restoration of invasion-resistant plant communities
Source: Commun Biol. 2023 Oct 30;6:1100. doi: 10.1038/s42003-023-05485-8 (PMC10616274; doi:10.1038/s42003-023-05485-8)

Supplementary information

Meta-analysis identifies native priority as a mechanism that supports the restoration of invasion-resistant plant communities

Melinda Halassy[1,2], Péter Batáry[1,3], Anikó Csecserits[1,2], Katalin Török[1], Orsolya Valkó[1,4]

[1] National Laboratory for Health Security, Centre for Ecological Research, 29 Karolina Street, H-1113 Budapest, Hungary

[2] Institute of Ecology and Botany, Centre for Ecological Research, 2-4 Alkotmány Street, H-2163 Vácrátót, Hungary

[3] 'Lendület' Landscape and Conservation Ecology Research Group, Institute of Ecology and Botany, Centre for Ecological Research, 2-4 Alkotmány Street, H-2163 Vácrátót, Hungary

[4] 'Lendület' Seed Ecology Research Group, Institute of Ecology and Botany, Centre for Ecological Research, 2-4 Alkotmány Street, H-2163 Vácrátót, Hungary

* Corresponding author: Melinda Halassy (halassy.melinda@ecolres.hu)

Supplementary Table 1. Study inclusion criteria according to the PICO-model. IAS: invasive alien species

**Population**	**Experimental restoration studies conducted in terrestrial plant communities**			
**Intervention**	Active introduction of native species and IAS via seeding or planting with manipulation of			
	functional similarity, seeding density and priority effect (focal study)	functional similarity	seeding density	priority effect
**Comparator**	control: only IAS	control: IAS + low similarity native species	control: IAS + low density native species	control: concurrent seeding of IAS + native species
	treatment: IAS + high similarity, high density, high priority native species	treatment: IAS + high similarity native species	treatment: IAS + high density native species	treatment: native priority + IAS
**Outcome**	Establishment of IAS (e.g. seedling emergence and survival)<br><br>Growth of IAS (e.g. percentage cover, biomass)			

Supplementary Table 2. Statistical results of focal model.

moderator	group	estimate	se	zval	pval	ci.lb	ci.ub
none	none	-0.4193	0.1787	-2.3462	0.0190	-0.7697	-0.0690
mechanism	density	-0.7678	0.2694	-2.8500	0.0044	-1.2958	-0.2398
	multiple	0.6747	0.7725	0.8733	0.3825	-0.8395	2.1888
	priority	-0.8259	0.2031	-4.0673	<.0001	-1.2239	-0.4279
	similarity	0.3340	0.2652	1.2595	0.2079	-0.1858	0.8539
indicator	establishment	-0.4557	0.1789	-2.5480	0.0108	-0.8063	-0.1052
	growth	0.0392	0.0091	4.3172	<.0001	0.0214	0.0570
life form	annual	-0.3083	0.1842	-1.6739	0.0942	-0.6693	0.0527
	perennial	-0.4856	0.1785	-2.7203	0.0065	-0.8354	-0.1357
functional type	grass	-0.4182	0.2026	-2.0644	0.0390	-0.8153	-0.0212
	non-grass	-0.3125	0.2051	-1.5237	0.1276	-0.7145	0.0895
experiment type	field	-0.2505	0.3651	-0.6862	0.4926	-0.9660	0.4650
	greenhouse	-0.3986	0.2635	-1.5125	0.1304	-0.9151	0.1179
	outdoor	-0.6402	0.3714	-1.7238	0.0847	-1.3681	0.0877
experiment length	between 6 months and 1 year	-0.2243	0.5218	-0.4299	0.6672	-1.2471	0.7984
	less than 6 months	-0.5305	0.2448	-2.1672	0.0302	-1.0103	-0.0507
	more than 1 year	-0.2923	0.3397	-0.8605	0.3895	-0.9581	0.3735

Supplementary Table 3. Post-hoc test outputs for mechanism as moderator in the focal model. Tukey test was applied to the model if there was a significant difference, and more than two groups were involved. 1 – seeding density, 2 – multiple mechanisms, 3 –priority effect, 4 – functional similarity. Signif. codes: 0 ‘***’, 0.001 ‘**’, 0.01 ‘*’ 0.05 ‘.’.

<table>
<tr><th></th><th>Estimate</th><th>Std. Error</th><th>z value</th><th>Pr(>|z|)</th></tr>
<tr><td>2 - 1 == 0</td><td>2.2102</td><td>0.9419</td><td>2.347</td><td>0.018945 *</td></tr>
<tr><td>3 - 1 == 0</td><td>0.7096</td><td>0.5106</td><td>1.390</td><td>0.164560</td></tr>
<tr><td>4 - 1 == 0</td><td>1.8696</td><td>0.5492</td><td>3.404</td><td>0.000664 ***</td></tr>
<tr><td>3 - 2 == 0 -</td><td>-1.5005</td><td>0.7988</td><td>-1.879</td><td>0.060305</td></tr>
<tr><td>4 - 2 == 0</td><td>-0.3406</td><td>0.8168</td><td>-0.417</td><td>0.676667</td></tr>
<tr><td>4 - 3 == 0</td><td>1.1599</td><td>0.3231</td><td>3.590</td><td>0.000330 ***</td></tr>
</table>

Supplementary Table 4. Statistical results of functional similarity model.

moderator	group	estimate	se	zval	pval	ci.lb	ci.ub
none	none	0.0194	0.1450	0.1339	0.8935	-0.2648	0.3036
indicator	establishment	0.8004	0.3064	2.6127	0.0090	0.2000	1.4008
	growth	0.0167	0.1454	0.1151	0.9083	-0.2683	0.3018
life form	annual	0.1320	0.2012	0.6561	0.5118	-0.2623	0.5263
	perennial	-0.0602	0.1752	-0.3434	0.7313	-0.4037	0.2833
functional type	grass	-0.0546	0.2359	-0.2317	0.8168	-0.5170	0.4077
	non-grass	0.0671	0.1979	0.3391	0.7346	-0.3209	0.4551
experiment type	field	-0.1298	0.2406	-0.5395	0.5895	-0.6013	0.3417
	greenhouse	0.1283	0.2243	0.5721	0.5672	-0.3113	0.5680
	outdoor	0.0819	0.5128	0.1597	0.8731	-0.9231	1.0870
experiment length	between 6 months and 1 year	0.7303	0.4453	1.6399	0.1010	-0.1425	1.6030
	less than 6 months	-0.0146	0.2243	-0.0650	0.9482	-0.4541	0.4250
	more than 1 year	-0.0844	0.1934	-0.4364	0.6625	-0.4634	0.2946

Supplementary Table 5. Statistical results of the seeding density model.

moderator	group	estimate	se	zval	pval	ci.lb	ci.ub
none	none	-0.1294	0.1883	-0.6870	0.4921	-0.4985	0.2397
indicator	establishment	0.0036	0.5069	0.0071	0.9944	-0.9899	0.9971
	growth	-0.1624	0.2271	-0.7151	0.4745	-0.6076	0.2828
life form	annual	-0.0309	0.1933	-0.1599	0.8730	-0.4097	0.3479
	perennial	-0.1946	0.1858	-1.0473	0.2950	-0.5587	0.1696
functional type	grass	0.0145	0.2432	0.0598	0.9523	-0.4620	0.4911
	non-grass	-0.3676	0.3112	-1.1811	0.2375	-0.9776	0.2424
experiment type	field	0.1218	0.1492	0.8162	0.4144	-0.1706	0.4142
	greenhouse	0.1492	0.1363	1.0947	0.2736	-0.1179	0.4163
	outdoor	-0.7608	0.1649	-4.6129	<.0001	-1.0841	-0.4376
experiment length	between 6 months and 1 year	0.0766	0.4912	0.1560	0.8761	-0.8862	1.0394
	less than 6 months	-0.3812	0.2810	-1.3564	0.1750	-0.9320	0.1696
	more than 1 year	0.1528	0.3543	0.4312	0.6664	-0.5417	0.8473

Supplementary Table 6. Post-hoc test outputs for mechanism as moderator in the seeding density model. Tukey test was applied to the model if there was a significant difference, and more than two groups were involved. 1 – seeding density, 2 – multiple mechanisms, 3 –priority effect, 4 – functional similarity. Signif. codes: 0 ‘***’, 0.001 ‘**’, 0.01 ‘*’ 0.05 ‘.’.

<table>
<tr><th></th><th>Estimate</th><th>Std. Error</th><th>z value</th><th>Pr(>|z|)</th></tr>
<tr><td>2 - 1 == 0</td><td>-0.09435</td><td>0.32802</td><td>-0.288</td><td>0.77362</td></tr>
<tr><td>3 - 1 == 0</td><td>-1.00435</td><td>0.34093</td><td>-2.946</td><td>0.00322 **</td></tr>
<tr><td>3 - 2 == 0</td><td>-0.90999</td><td>0.21395</td><td>-4.253</td><td>2.11e-05 ***</td></tr>
</table>

Supplementary Table 7. Statistical results of priority effect model.

moderator	group	estimate	se	zval	pval	ci.lb	ci.ub
none	none	-1.0766	0.2932	-3.6721	0.0002	-1.6512	-0.5020
indicator	establishment	-1.0968	0.2937	-3.7351	0.0002	-1.6724	-0.5213
	growth	-1.0759	0.2935	-3.6664	0.0002	-1.6511	-0.5008
life form	annual	-0.9246	0.2871	-3.2208	0.0013	-1.4872	-0.3619
	perennial	-1.1700	0.2828	-4.1368	<.0001	-1.7244	-0.6157
functional type	grass	-1.0926	0.3185	-3.4308	0.0006	-1.7168	-0.4684
	non-grass	-0.8941	0.3343	-2.6748	0.0075	-1.5492	-0.2389
experiment type	field	-0.8970	0.7440	-1.2056	0.2280	-2.3552	0.5613
	greenhouse	-1.0244	0.4446	-2.3042	0.0212	-1.8958	-0.1530
	outdoor	-1.3490	0.6180	-2.1830	0.0290	-2.5601	-0.1378
experiment length	between 6 months and 1 year	-0.1266	0.4624	-0.2739	0.7842	-1.0328	0.7796
	less than 6 months	-1.4701	0.3237	-4.5415	<.0001	-2.1045	-0.8357
	more than 1 year	-1.2937	0.8032	-1.6108	0.1072	-2.8679	0.2804

Supplementary Table 8. Statistical tests for checking publication bias. Kendall’s Rank Correlation Test for Funnel Plot Asymmetry and Egger’s test for significant sampling bias. Signif. codes: 0 ‘***’, 0.05 ‘.’

	Kendall’s test		Egger’s test	
Model	tau	p	z value	p
Focal study	0.0828	0.2532	-1.6252	0.1041
Functional similarity	-0.0100	0.9337	2.3125	0.0207.
Seeding density	-0.3099	0.0684	-4.1036	<0.0001***
Priority effect	-0.1613	0.2104	0.0401	0.9680

Supplementary Note 1. Details of meta-analysis models described with model formula in R-syntax. Results are presented in Table 1 and Figure 2-5.

Summary random-effect model without moderator (referred to as "none" in tables and figures)

rma.mv(yi=lnRR,vi= Var_lnRR, method="REML", random=list(~1|StudyID))

Example of analysis with moderator (life form of invasive alien species)

rma.mv(yi=lnRR,vi= Var_lnRR, mods=~life_form, method="REML", random=list(~1|StudyID))

Supplementary Note 2. Model performance with and without outliers as measured by AICc. Smaller values indicate better performing models.

2.1. a. Full model with outliers:

```
 Multivariate Meta-Analysis Model (k = 98; method: REML)

   logLik   Deviance        AIC        BIC       AICc
-319.9175   639.8351   643.8351   648.9845   643.9627

Variance Components:

            estim    sqrt  nlvls  fixed   factor
sigma^2    3.1734  1.7814     26     no  StudyID

Test for Heterogeneity:
Q(df = 97) = 2644.3003, p-val < .0001

Model Results:

estimate      se     zval    pval    ci.lb    ci.ub
 -0.7889  0.3517  -2.2432  0.0249  -1.4783  -0.0996  *
```

2.1.b. Full model without outliers:

```
Multivariate Meta-Analysis Model (k = 88; method: REML)

   logLik   Deviance        AIC        BIC       AICc
-103.1925   206.3850   210.3850   215.3168   210.5278

Variance Components:

            estim    sqrt  nlvls  fixed   factor
sigma^2    0.7296  0.8542     24     no  StudyID

Test for Heterogeneity:
Q(df = 87) = 1091.1517, p-val < .0001

Model Results:

estimate      se     zval    pval    ci.lb    ci.ub
 -0.4193  0.1787  -2.3462  0.0190  -0.7697  -0.0690  *
```

2.2. a. Functional similarity model with outliers:

```
Multivariate Meta-Analysis Model (k = 43; method: REML)

   logLik   Deviance        AIC        BIC       AICc
-114.7486   229.4973   233.4973   236.9726   233.8050

Variance Components:

            estim    sqrt  nlvls  fixed   factor
sigma^2    1.1162  1.0565     13     no  StudyID

Test for Heterogeneity:
Q(df = 42) = 775.7534, p-val < .0001

Model Results:

estimate      se    zval    pval    ci.lb   ci.ub
  0.3163  0.2993  1.0566  0.2907  -0.2704  0.9029
```

2.2. b. Functional similarity model with outliers:

```
Multivariate Meta-Analysis Model (k = 38; method: REML)

  logLik  Deviance       AIC       BIC      AICc
-51.3122  102.6244  106.6244  109.8462  106.9773

Variance Components:

            estim    sqrt  nlvls  fixed   factor
sigma^2    0.2120  0.4605     12     no  StudyID

Test for Heterogeneity:
Q(df = 37) = 251.9482, p-val < .0001

Model Results:

estimate      se    zval    pval    ci.lb   ci.ub
  0.0194  0.1450  0.1339  0.8935  -0.2648  0.3036
```

2.3. a. Seeding density model with outliers:

```
Multivariate Meta-Analysis Model (k = 21; method: REML)

  logLik  Deviance       AIC       BIC      AICc
-49.4702   98.9404  102.9404  104.9319  103.6463

Variance Components:

            estim    sqrt  nlvls  fixed   factor
sigma^2    0.2084  0.4565      7     no  StudyID

Test for Heterogeneity:
Q(df = 20) = 563.5029, p-val < .0001

Model Results:

estimate      se     zval    pval    ci.lb   ci.ub
 -0.1298  0.1884  -0.6886  0.4910  -0.4991  0.2395
```

2.3.b. Seeding density without outliers:

```
Multivariate Meta-Analysis Model (k = 19; method: REML)

  logLik  Deviance       AIC       BIC      AICc
-43.1515   86.3029   90.3029   92.0837   91.1029

Variance Components:

            estim    sqrt  nlvls  fixed   factor
sigma^2    0.2081  0.4562      7     no  StudyID

Test for Heterogeneity:
Q(df = 18) = 563.1752, p-val < .0001

Model Results:

estimate      se     zval    pval    ci.lb   ci.ub
 -0.1294  0.1883  -0.6870  0.4921  -0.4985  0.2397
```

2.4. a. Priority effect model with outliers:

```
Multivariate Meta-Analysis Model (k = 31; method: REML)

  logLik  Deviance       AIC       BIC      AICc
-76.7076  153.4151  157.4151  160.2175  157.8596

Variance Components:

            estim    sqrt  nlvls  fixed   factor
sigma^2    1.2395  1.1133     11     no  StudyID

Test for Heterogeneity:
Q(df = 30) = 787.1347, p-val < .0001

Model Results:

estimate      se     zval    pval    ci.lb    ci.ub
 -1.1911  0.3417  -3.4858  0.0005  -1.8608  -0.5214  ***
```

2.4.b. Priority effect without outliers:

```
Multivariate Meta-Analysis Model (k = 29; method: REML)

  logLik  Deviance       AIC       BIC      AICc
-71.9212  143.8423  147.8423  150.5067  148.3223

Variance Components:

            estim    sqrt  nlvls  fixed   factor
sigma^2    0.8740  0.9349     11     no  StudyID

Test for Heterogeneity:
Q(df = 28) = 703.8589, p-val < .0001

Model Results:

estimate      se     zval    pval    ci.lb    ci.ub
 -1.0766  0.2932  -3.6721  0.0002  -1.6512  -0.5020  ***
```

Supplementary Figure 1. PRISMA flow chart detailing the selection of studies included in the review and meta-analyses. After MJ, McKenzie JE, Bossuyt PM, Boutron I, Hoffmann TC, Mulrow CD, et al. The PRISMA 2020 statement: an updated guideline for reporting systematic reviews. BMJ 2021;372:n71. doi: 10.1136/bmj.n71 For more information, visit: http://www.prisma-statement.org/

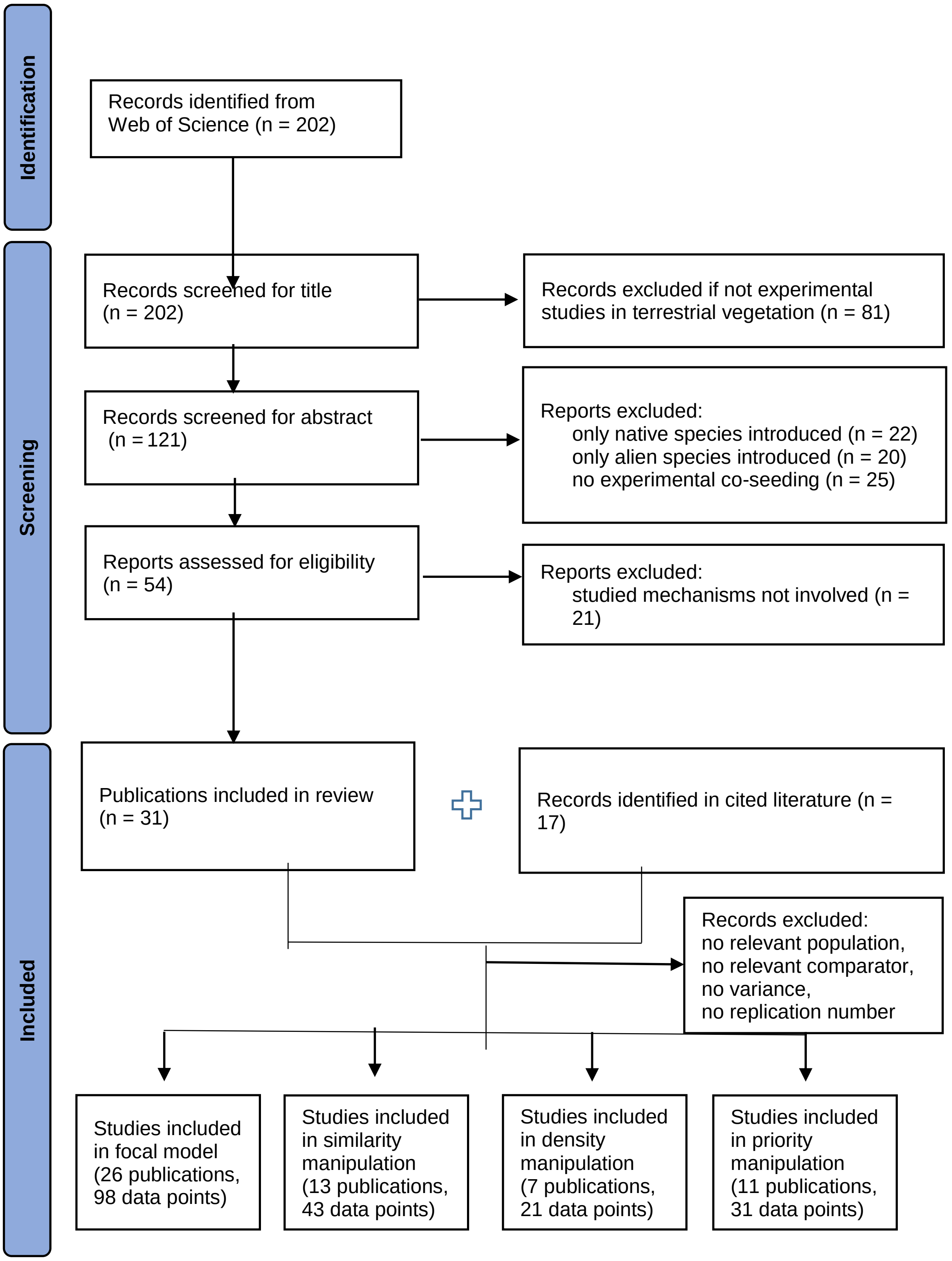

Supplementary Figure 2. Funnel plots showing the relationship between effect sizes and standard error for the summary meta-analyses without moderators. (a) focal model (n=26, k=98), (b) functional similarity model (n=, 13, k=43), (c) seeding density model (n=7, k=20), (d) priority effect model (n=11, k=31). The true effect size is indicated by the dotted line.

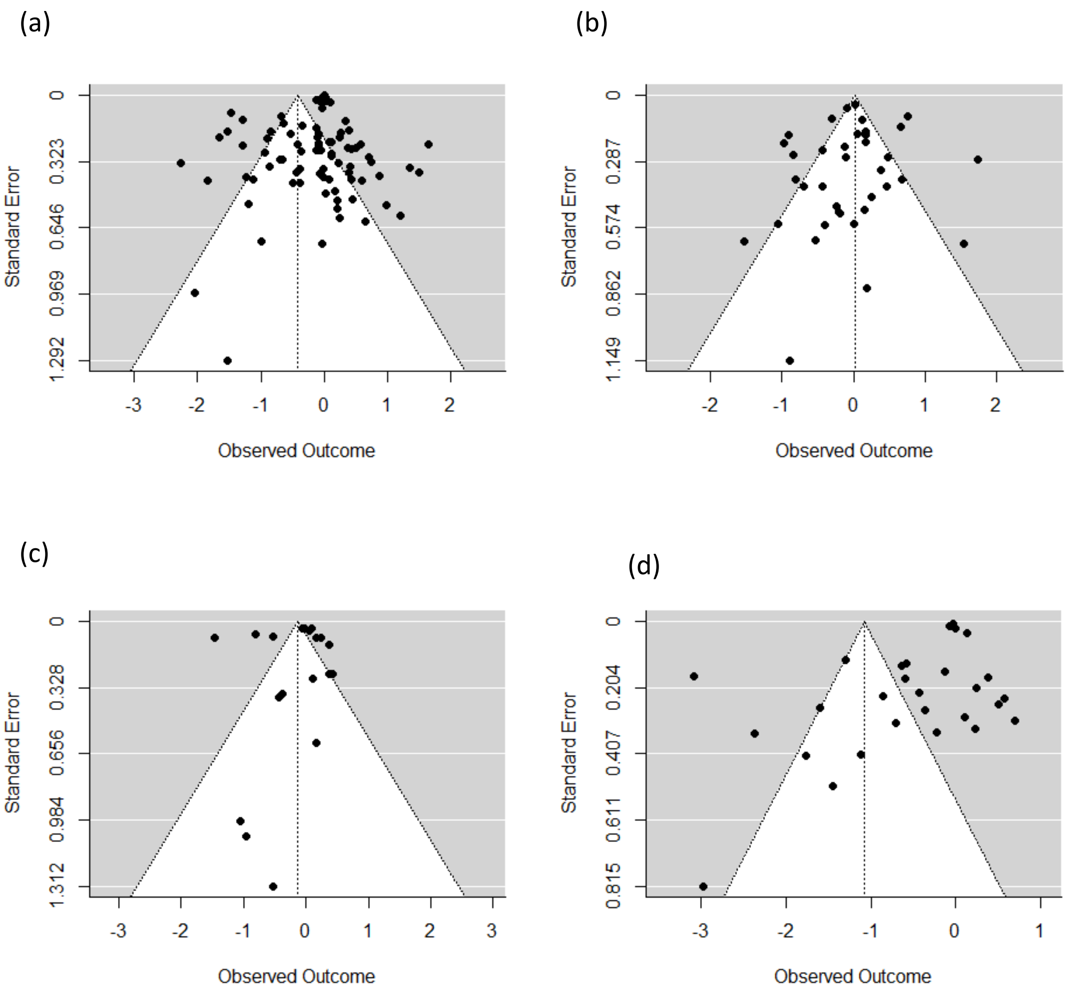

Supplement: Supplementary file 1 — Supplementary information [file 42003_2023_5485_MOESM1_ESM.pdf]
