# Supplementary material for: Meta-analysis identifies native priority as a mechanism that supports the restoration of invasion-resistant plant communities
Source: Commun Biol. 2023 Oct 30;6:1100. doi: 10.1038/s42003-023-05485-8 (PMC10616274; doi:10.1038/s42003-023-05485-8)
Supplement: Supplementary file 2 — Description of Additional Supplementary Files [file 42003_2023_5485_MOESM2_ESM.pdf]

Description of Additional Supplementary Files

File name: Supplementary Data 1
Description: Basic data of the selected publications and references for the four model in text format.

File name: Supplementary Data 2
Description: Results of test statistics that is the source data behind Figure 2-5 in xls format.
