# Supplementary material for: Meta-analysis identifies native priority as a mechanism that supports the restoration of invasion-resistant plant communities
Source: Commun Biol. 2023 Oct 30;6:1100. doi: 10.1038/s42003-023-05485-8 (PMC10616274; doi:10.1038/s42003-023-05485-8)
Supplement: Supplementary file 3 — Supplementary Data 1 [file 42003_2023_5485_MOESM3_ESM.rtf]

Supplementary Data 1: Basic data of the selected publications and resulting data points and references for the four model used in
Meta-analysis identifies native priority as a mechanism that supports the restoration of invasion-resistant plant communities
Melinda Halassy1,2, Péter Batáry1,3, Anikó Csecserits1,2, Katalin Török1, Orsolya Valkó1,4
1 National Laboratory for Health Security, Centre for Ecological Research, 29 Karolina Street, H-1113 Budapest, Hungary
2 Institute of Ecology and Botany, Centre for Ecological Research, 2-4 Alkotmány Street, H-2163 Vácrátót, Hungary
3 'Lendület' Landscape and Conservation Ecology Research Group, Institute of Ecology and Botany, Centre for Ecological Research, 2-4 Alkotmány Street, H-2163 Vácrátót, Hungary
4 'Lendület' Seed Ecology Research Group, Institute of Ecology and Botany, Centre for Ecological Research, 2-4 Alkotmány Street, H-2163 Vácrátót, Hungary
* Corresponding author: Melinda Halassy (halassy.melinda@ecolres.hu)

Supplementary Data 1a. Basic data of the selected publications (n=26) and resulting data points (k=98) of the focal model. The focal model included all three mechanisms (functional similarity, seeding density and priority effect) where seeding of invasive species without native species was considered as control. Effect size used was log response ration (lnRR). Studies involved into the model are listed below. * Excluded outliers. ** Excluded for high variance.
Source	Country	Studied mechanism	Performance indicator	Invasive alien species	Life form	Functional group	Experiment type	Experiment lenght	Number of samples	lnRR	var (lnRR)	
Brisson et al. (2020)	Canada	functional similarity	establishment	Heracleum mantegazzianum	perennial	non-grass	field	more than 1 year	8	-1.2315	0.1575	
Brisson et al. (2020)	Canada	functional similarity	establishment	Heracleum mantegazzianum	perennial	non-grass	field	more than 1 year	8	-0.6640	0.0975	
Brisson et al. (2020)	Canada	functional similarity	establishment	Heracleum mantegazzianum	perennial	non-grass	field	more than 1 year	8	-0.8679	0.1206	
Brisson et al. (2020)	Canada	functional similarity	growth	Heracleum mantegazzianum	perennial	non-grass	field	more than 1 year	8	-0.9115	0.0443	
Brisson et al. (2020)	Canada	functional similarity	growth	Heracleum mantegazzianum	perennial	non-grass	field	more than 1 year	8	-0.8478	0.0302	
Brisson et al. (2020)	Canada	functional similarity	growth	Heracleum mantegazzianum	perennial	non-grass	field	more than 1 year	8	-0.0294	0.0036	
Byun & Lee (2017)*	South Korea	functional similarity	growth	Ageratina altissima	perennial	non-grass	greenhouse	less than 6 months	3	1.3486	0.1240	
Byun & Lee (2017)*	South Korea	functional similarity	growth	Ageratina altissima	perennial	non-grass	greenhouse	less than 6 months	3	0.9808	0.2874	
Byun & Lee (2017)	South Korea	functional similarity	growth	Ageratina altissima	perennial	non-grass	greenhouse	less than 6 months	3	0.6371	0.3804	
Byun et al. (2013)	USA	functional similarity	growth	Phragmites australis	perennial	grass	outdoor	more than 1 year	3	1.6358	0.0579	
Byun et al. (2013)*	USA	functional similarity	growth	Phragmites australis	perennial	grass	outdoor	more than 1 year	3	3.6507	0.0699	
Byun et al. (2013)	USA	functional similarity	growth	Phragmites australis	perennial	grass	outdoor	more than 1 year	3	1.2083	0.3459	
Byun et al. (2013)	USA	functional similarity	growth	Phragmites australis	perennial	grass	outdoor	more than 1 year	3	0.7465	0.1067	
Byun et al. (2013)	USA	functional similarity	growth	Phragmites australis	perennial	grass	outdoor	more than 1 year	3	0.5826	0.1713	
Byun et al. (2020)	South Korea	functional similarity	growth	Sicyos angulatus	annual	non-grass	greenhouse	less than 6 months	3	0.4474	0.2574	
Byun et al. (2020)	South Korea	functional similarity	growth	Sicyos angulatus	annual	non-grass	greenhouse	less than 6 months	3	0.4231	0.1676	
Byun et al. (2020)	South Korea	functional similarity	growth	Sicyos angulatus	annual	non-grass	greenhouse	less than 6 months	3	0.0696	0.1676	
Byun et al. (2020)	South Korea	functional similarity	growth	Sicyos angulatus	annual	non-grass	greenhouse	less than 6 months	3	-0.0216	0.1569	
Byun et al. (2020)	South Korea	functional similarity	growth	Sicyos angulatus	annual	non-grass	greenhouse	less than 6 months	12	0.2076	0.2639	
Byun et al. (2020)	South Korea	seeding density	growth	Sicyos angulatus	annual	non-grass	greenhouse	less than 6 months	3	-0.9954	0.5090	
Byun et al. (2020)**	South Korea	seeding density	growth	Sicyos angulatus	annual	non-grass	greenhouse	less than 6 months	3	-1.5261	1.6687	
Byun et al. (2020)	South Korea	seeding density	growth	Sicyos angulatus	annual	non-grass	greenhouse	less than 6 months	3	-2.0369	0.9232	
Cleland et al. (2015)	USA	priority effect	growth	Bromus hordeaceus	annual	grass	outdoor	less than 6 months	8	-0.1226	0.0242	
Cleland et al. (2015)	USA	priority effect	growth	Trifolium hirtum	annual	non-grass	outdoor	less than 6 months	8	0.2255	0.1089	
Cleland et al. (2015)	USA	priority effect	growth	Trifolium hirtum	annual	non-grass	outdoor	less than 6 months	8	0.1112	0.0864	
Cleland et al. (2015)	USA	priority effect	growth	Bromus hordeaceus	annual	grass	outdoor	less than 6 months	8	0.5067	0.0643	
Cleland et al. (2015)	USA	priority effect	growth	Bromus hordeaceus	annual	grass	outdoor	less than 6 months	8	0.3827	0.0294	
Cleland et al. (2015)	USA	priority effect	growth	Bromus hordeaceus	annual	grass	outdoor	less than 6 months	8	-0.6995	0.0985	
Cleland et al. (2015)	USA	priority effect	growth	Lolium multiflorum	perennial	grass	outdoor	less than 6 months	8	-0.6423	0.0184	
Cleland et al. (2015)	USA	priority effect	growth	Lolium multiflorum	perennial	grass	outdoor	less than 6 months	8	0.5733	0.0557	
Cleland et al. (2015)	USA	priority effect	growth	Lolium multiflorum	perennial	grass	outdoor	less than 6 months	8	0.6931	0.0920	
Cleland et al. (2015)	USA	priority effect	growth	Lolium multiflorum	perennial	grass	outdoor	less than 6 months	8	0.2451	0.0423	
Delory et al. (2019)	Germany	priority effect	growth	Senecio inaequidens	perennial	non-grass	greenhouse	less than 6 months	5	-3.6889	0.1597	
Delory et al. (2019)	Germany	priority effect	growth	Senecio inaequidens	perennial	non-grass	greenhouse	less than 6 months	5	-2.9755	0.6637	
Evangelista et al. (2017)	Brazil	priority effect	growth	Urochloa arrecta	perennial	grass	greenhouse	less than 6 months	6	-1.5363	0.0300	
Firn et al. (2010)*	Australia	multiple	growth	Eragrostis curvula	perennial	grass	greenhouse	between 6 months and 1 year	3	-7.9178	0.5694	
Firn et al. (2010)*	Australia	multiple	growth	Eragrostis curvula	perennial	grass	greenhouse	between 6 months and 1 year	3	-6.7123	0.7593	
Firn et al. (2010)*	Australia	multiple	growth	Eragrostis curvula	perennial	grass	greenhouse	between 6 months and 1 year	3	-6.8859	0.5580	
Grman& Suding (2010)	USA	priority effect	growth	several species	annual	NA	outdoor	less than 6 months	7	-1.8405	0.1714	
Hess et al. (2020)	France	priority effect	establishment	Ambrosia artemisiifolia	annual	non-grass	greenhouse	between 6 months and 1 year	5	-0.0499	0.0003	
Hess et al. (2020)	France	priority effect	growth	Cortaderia selloana	annual	non-grass	greenhouse	between 6 months and 1 year	5	-0.0045	0.0001	
Hess et al. (2020)	France	priority effect	establishment	Ambrosia artemisiifolia	annual	non-grass	greenhouse	between 6 months and 1 year	5	-0.1239	0.0004	
Hess et al. (2020)	France	priority effect	establishment	Bothriochloa barbinodis	annual	non-grass	greenhouse	between 6 months and 1 year	5	-0.0059	0.0007	
Hess et al. (2020)	France	priority effect	establishment	Bothriochloa barbinodis	annual	non-grass	greenhouse	between 6 months and 1 year	5	-0.0133	0.0006	
Hess et al. (2020)	France	priority effect	establishment	Cortaderia selloana	annual	non-grass	greenhouse	between 6 months and 1 year	5	0.0511	0.0006	
Hess et al. (2020)	France	priority effect	establishment	Cortaderia selloana	annual	non-grass	greenhouse	between 6 months and 1 year	5	0.0068	0.0006	
Hess et al. (2020)	France	priority effect	growth	Ambrosia artemisiifolia	annual	non-grass	greenhouse	between 6 months and 1 year	5	0.0022	0.0000	
Hess et al. (2020)	France	priority effect	growth	Ambrosia artemisiifolia	annual	non-grass	greenhouse	between 6 months and 1 year	5	0.0011	0.0000	
Hess et al. (2020)	France	priority effect	growth	Cortaderia selloana	annual	non-grass	greenhouse	between 6 months and 1 year	5	0.0244	0.0001	
Lang et al. (2017)	Germany	priority effect	growth	Cytisus scoparius	perennial	non-grass	greenhouse	between 6 months and 1 year	15	-0.9460	0.0787	
Mason et al. (2013)	USA	priority effect	growth	Chrysanthemoides monilifera ssp. rotundata	perennial	non-grass	outdoor	more than 1 year	5	-1.2873	0.0609	
Mason et al. (2013)	USA	priority effect	establishment	Chrysanthemoides monilifera ssp. rotundata	perennial	non-grass	outdoor	more than 1 year	5	-1.6672	0.0426	
Park et al. (2022)	South Korea	functional similarity	growth	 Festuca arundinacea	perennial	grass	greenhouse	less than 6 months	3	-0.0846	0.0703	
Park et al. (2022)	South Korea	functional similarity	growth	 Festuca arundinacea	perennial	grass	greenhouse	less than 6 months	3	-0.0664	0.1460	
Park et al. (2022)	South Korea	functional similarity	growth	 Festuca arundinacea	perennial	grass	greenhouse	less than 6 months	3	-0.1372	0.0703	
Park et al. (2022)	South Korea	functional similarity	growth	 Festuca arundinacea	perennial	grass	greenhouse	less than 6 months	3	-0.0479	0.0703	
Park et al. (2022)	South Korea	functional similarity	growth	 Festuca arundinacea	perennial	grass	greenhouse	less than 6 months	3	0.0817	0.0523	
Park et al. (2022)	South Korea	functional similarity	growth	 Festuca arundinacea	perennial	grass	greenhouse	less than 6 months	3	-0.0846	0.0346	
Park et al. (2022)	South Korea	functional similarity	growth	 Festuca arundinacea	perennial	grass	greenhouse	less than 6 months	3	0.1032	0.0523	
Perkins & Hatfield (2014)	USA	priority effect	growth	Agropyron cristatum	perennial	grass	greenhouse	less than 6 months	9	-0.0964	0.0609	
Perkins & Hatfield (2014)	USA	priority effect	growth	Bromus tectorum	annual	grass	greenhouse	less than 6 months	9	-0.4898	0.1837	
Perkins & Hatfield (2014)	USA	priority effect	growth	Taeniatherum caput-medusae 	annual	grass	greenhouse	less than 6 months	9	-0.3878	0.1837	
Schantz et al. (2015)	USA	seeding density	growth	Bromus tectorum	annual	grass	field	more than 1 year	3	0.1030	0.0802	
Schantz et al. (2015)	USA	seeding density	growth	Bromus tectorum	annual	grass	field	more than 1 year	3	0.3766	0.0662	
Schantz et al. (2015)	USA	seeding density	growth	Bromus tectorum	annual	grass	field	more than 1 year	3	0.4335	0.0669	
Schantz et al. (2019)	USA	seeding density	establishment	Taniatherum caput-medusae, Bromus tectorum	annual	grass	field	more than 1 year	3	-0.0290	0.0012	
Schantz et al. (2019)	USA	seeding density	establishment	Taniatherum caput-medusae, Bromus tectorum	annual	grass	field	more than 1 year	3	0.0910	0.0011	
Schantz et al. (2019)	USA	seeding density	establishment	Taniatherum caput-medusae, Bromus tectorum	annual	grass	field	more than 1 year	3	-0.0588	0.0012	
Stevens & Fehmi (2011)	USA	priority effect	growth	Pennisetum ciliare	perennial	grass	greenhouse	less than 6 months	5	-2.2736	0.1091	
Tarsa et al. (2022)	USA	seeding density	growth	Phalaris arundinacea 	perennial	grass	outdoor	less than 6 months	6	-0.3782	0.1294	
Tarsa et al. (2022)	USA	seeding density	growth	Phalaris arundinacea 	perennial	grass	outdoor	less than 6 months	6	-0.4415	0.1397	
Tarsa et al. (2022)	USA	priority effect	growth	Phalaris arundinacea 	perennial	grass	outdoor	less than 6 months	6	-0.3610	0.0750	
Tarsa et al. (2022)	USA	priority effect	growth	Phalaris arundinacea 	perennial	grass	outdoor	less than 6 months	6	-3.0910	0.0278	
Ulrich & Perkins (2014)	USA	priority effect	growth	Bromus inermis	perennial	grass	greenhouse	less than 6 months	10	-2.3775	0.1179	
Ulrich & Perkins (2014)	USA	priority effect	growth	Poa pratensis	perennial	grass	greenhouse	less than 6 months	10	-1.1206	0.1685	
Vaughn & Young (2015)	USA	priority effect	growth	various species	annual	grass	field	more than 1 year	5	-0.5288	0.0344	
Wohlwend et al. (2019)	USA	priority effect	growth	Lespedeza cuneata	perennial	non-grass	field	more than 1 year	51	-1.2937	0.0139	
Yannelli et al. *(2017a)	Germany	similarity*density	growth	Solidago gigantea	perennial	non-grass	greenhouse	less than 6 months	5	4.5326	0.0367	
Yannelli et al. (2017a)	Germany	similarity*density	growth	Solidago gigantea	perennial	non-grass	greenhouse	less than 6 months	5	1.4881	0.1418	
Yannelli et al. (2017a)	Germany	similarity*density	growth	Ambrosia artemisiifolia	annual	non-grass	greenhouse	less than 6 months	5	0.2353	0.3575	
Yannelli et al. (2017a)	Germany	similarity*density	growth	Ambrosia artemisiifolia	annual	non-grass	greenhouse	less than 6 months	5	0.0108	0.2286	
Yannelli et al. (2017a)	Germany	similarity*density	growth	Solidago gigantea	perennial	non-grass	greenhouse	less than 6 months	5	1.8971	0.0618	
Yannelli et al. (2017a)	Germany	similarity*density	growth	Solidago gigantea	perennial	non-grass	greenhouse	less than 6 months	5	0.8726	0.1552	
Yannelli et al. (2017a)	Germany	similarity*density	growth	Ambrosia artemisiifolia	annual	non-grass	greenhouse	less than 6 months	5	0.2054	0.3059	
Yannelli et al. (2017a)	Germany	similarity*density	growth	Ambrosia artemisiifolia	annual	non-grass	greenhouse	less than 6 months	5	-0.0282	0.5230	
Yannelli et al. (2018)	Germany	functional similarity	growth	Ambrosia artemisiifolia	annual	non-grass	greenhouse	less than 6 months	6	-0.3438	0.0221	
Yannelli et al. (2018)	Germany	functional similarity	growth	Solidago gigantea	perennial	non-grass	greenhouse	less than 6 months	6	-0.6763	0.0098	
Yannelli et al. (2017b)	Germany	functional similarity	growth	Ambrosia artemisiifolia	annual	non-grass	greenhouse	less than 6 months	6	-0.0230	0.1295	
Yannelli et al. (2017b)	Germany	functional similarity	growth	Nassella trichotoma	annual	non-grass	greenhouse	less than 6 months	6	0.3979	0.1190	
Yannelli et al. (2017b)	Germany	functional similarity	growth	Solidago gigantea	perennial	non-grass	greenhouse	less than 6 months	6	-0.1112	0.0427	
Yannelli et al. (2017b)	Germany	functional similarity	growth	Solidago gigantea	perennial	non-grass	greenhouse	less than 6 months	6	-0.4321	0.0574	
Young et al. (2014)	USA	priority effect	growth	Vulpia (Festuca) myuros/V. bromoides, Bromus hordeaceus, Hordeum murinum, Avena barbata/A. fatua	annual	grass	field	between 6 months and 1 year	5	-1.1905	0.2824	
Young et al. (2014)	USA	priority effect	growth	Vulpia (Festuca) myuros/V. bromoides, Bromus hordeaceus, Hordeum murinum, Avena barbata/A. fatua	annual	grass	field	between 6 months and 1 year	5	0.2603	0.0322	
Young et al. (2014)	USA	priority effect	growth	Vulpia (Festuca) myuros/V. bromoides, Bromus hordeaceus, Hordeum murinum, Avena barbata/A. fatua	annual	grass	field	between 6 months and 1 year	5	0.3300	0.0153	
Young et al. (2014)	USA	priority effect	growth	Vulpia (Festuca) myuros/V. bromoides, Bromus hordeaceus, Hordeum murinum, Avena barbata/A. fatua	annual	grass	field	between 6 months and 1 year	5	-0.0946	0.0553	
Young et al. (2014)	USA	priority effect	growth	Vulpia (Festuca) myuros/V. bromoides, Bromus hordeaceus, Hordeum murinum, Avena barbata/A. fatua	annual	grass	field	between 6 months and 1 year	5	0.3942	0.1416	
Young et al. (2014)	USA	priority effect	growth	Vulpia (Festuca) myuros/V. bromoides, Bromus hordeaceus, Hordeum murinum, Avena barbata/A. fatua	annual	grass	field	between 6 months and 1 year	5	0.1653	0.2182	
Yu et al. (2020)	China	seeding density	growth	Mikania micrantha	perennial	non-grass	outdoor	less than 6 months	15	-1.4691	0.0066	

References of studies included in the focal model
Brisson, J., Teasdale, V., Boivin, P., & Lavoie, C. (2020). Plant cover restoration to inhibit seedling emergence, growth or survival of an exotic invasive plant species. Ecoscience, 27(3), 185-194. 
Byun, C., & Lee, E. J. (2017). Ecological application of biotic resistance to control the invasion of an invasive plant, Ageratina altissima. Ecology and evolution, 7(7), 2181-2192.
Byun, C., De Blois, S., & Brisson, J. (2013). Plant functional group identity and diversity determine biotic resistance to invasion by an exotic grass. Journal of Ecology, 101, 128–139 
Byun, C., Oh, M., Lee, E. J., & Kang, H. (2020). Seed density is as important as limiting similarity, diversity effect, and propagule pressure in plant restoration to control invasion. Ecological Engineering, 144, 105712.
Cleland, E. E., Esch, E., & McKinney, J. (2015). Priority effects vary with species identity and origin in an experiment varying the timing of seed arrival. Oikos, 124(1), 33-40.
Delory, B.M., Weidlich, E.W., Kunz, M., Neitzel, J. and Temperton, V.M. (2019) The exotic species Senecio inaequidens pays the price for arriving late in temperate European grassland communities. Oecologia, 191, 657-671. http://doi.org/10.1007/s00442-019-04521-x
Evangelista, H. B., Michelan, T. S., Gomes, L. C., & Thomaz, S. M. (2017). Shade provided by riparian plants and biotic resistance by macrophytes reduce the establishment of an invasive Poaceae. Journal of Applied Ecology, 54(2), 648-656.
Firn, J., MacDougall, A. S., Schmidt, S., & Buckley, Y. M. (2010). Early emergence and resource availability can competitively favour natives over a functionally similar invader. Oecologia, 163(3), 775-784.
Grman E & Suding KN (2010) Within-year soil legacies contribute to strong priority effects of exotics on native California grassland communities. Restoration Ecology 18, 664–670.
Hess, M. C. M., Buisson, E., Fontes, H., Bacon, L., Sabatier, F., & Mesléard, F. (2020). Giving recipient communities a greater head start and including productive species boosts early resistance to invasion. Applied Vegetation Science, 23(3), 340-352.
Lang, M., Hanslin, H. M., Kollmann, J., & Wagner, T. (2017). Suppression of an invasive legume by a native grass—High impact of priority effects. Basic and applied ecology, 22, 20-27.
Mason, T. J., French, K., & Jolley, D. (2013). Arrival order among native plant functional groups does not affect invasibility of constructed dune communities. Oecologia, 173(2), 557–568.
Park, S., Kim, J. H., & Lee, E. J. (2022). Resistance of plant communities to invasion by tall fescue: An experimental study combining species diversity, functional traits and nutrient levels. Basic and Applied Ecology, 58, 39-49.
Perkins, L. B., & Hatfield, G. (2014). Competition, legacy, and priority and the success of three invasive species. Biological invasions, 16(12), 2543-2550.
Schantz, M. C., Sheley, R. L., & James, J. J. (2015). Role of propagule pressure and priority effects on seedlings during invasion and restoration of shrub-steppe. Biological Invasions, 17(1), 73-85.
Schantz, M. C., Sheley, R. L., & James, J. J. (2019). Propagule pressure and priority seeding effects on the demography of invasive annual and native perennial grass species. Plant Ecology & Diversity, 12(2), 139-150.
Stevens, J. M., and J. S. Fehmi. 2011. Early establishment of a native grass reduces the competitive effect of a nonnative grass. Restoration Ecology 19:399–406
Tarsa, E. E., Holdaway, B. M., & Kettenring, K. M. (2022). Tipping the balance: The role of seed density, abiotic filters, and priority effects in seed?based wetland restoration. Ecological Applications, 32(8), e2706.
Ulrich E, Perkins L (2014) Bromus inermis and Elymus canadensis but not Poa pratensis demonstrate strong competitive effects and all benefit from priority. Plant Ecology 215:1269–1275
Vaughn, K.J. & Young, T.P. 2015. Short-term priority over exotic annuals increases the initial density and longer-term cover of native perennial grasses. Ecological Applications 25: 791– 799. 
Wohlwend, M. R., Schutzenhofer, M. R., & Knight, T. M. (2019). Long?term experiment manipulating community assembly results in favorable restoration outcomes for invaded prairies. Restoration Ecology, 27(6), 1307-1316.
Yannelli, F. A., Hughes, P., & Kollmann, J. (2017). Preventing plant invasions at early stages of revegetation: The role of limiting similarity in seed size and seed density. Ecological Engineering, 100, 286-290.
Yannelli, F. A., Karrer, G., Hall, R., Kollmann, J., & Heger, T. (2018). Seed density is more effective than multi?trait limiting similarity in controlling grassland resistance against plant invasions in mesocosms. Applied Vegetation Science, 21(3), 411-418.
Yannelli, F. A., Koch, C., Jeschke, J. M., and Kollmann, J. (2017b). Limiting similarity and Darwin's naturalization hypothesis: understanding the drivers of biotic resistance against invasive plant species. Oecologia 183, 775–784. doi: 10.1007/s00442-016-3798-8 
Young, T. P., Zefferman, E. P., Vaughn, K. J., & Fick, S. (2014). Initial success of native grasses is contingent on multiple interactions among exotic grass competition, temporal priority, rainfall and site effects. AoB Plants, 7, plu081
Yu, H., Yue, M., Wang, C., Le Roux, J. J., Peng, C., & Li, W. (2020). Priority effects and competition by a native species inhibit an invasive species and may assist restoration. Ecology and Evolution, 10(23), 13355-13369.


Supplementary Data 1b. Basic data of the selected publications (n=13) and resulting data points (k=43) of the functional similarity model. The functional similarity model considered seeding of low similarity native species as control and seeding of high-similarity native species as treatment. Effect size used was log response ration (lnRR). Studies involved into the model are listed below. * Excluded as outlier.
Source	Country	Studied mechanism	Performance indicator	Invasive alien species	Life form	Functional group	Experiment type	Experiment lenght	Number of samples	lnRR	var (lnRR)	
Allen & Meyer (2014)	USA	similarity	growth	Bromus tectorum	annual	grass	field	more	6	0.1643	0.0287	
Allen & Meyer (2014)	USA	similarity	growth	Aegilops cylindrica	annual	grass	field	more	6	0.1286	0.0109	
Allen & Meyer (2014)	USA	similarity	growth	Secale cereale	annual	grass	field	more	6	0.1643	0.0409	
Brisson et al. (2020)	Canada	similarity	establishment	Heracleum mantegazzianum	perennial	non-grass	field	more	8	0.6769	0.1310	
Brisson et al. (2020)	Canada	similarity	establishment	Heracleum mantegazzianum	perennial	non-grass	field	more	8	0.4730	0.1541	
Brisson et al. (2020)	Canada	similarity	growth	Heracleum mantegazzianum	perennial	non-grass	field	more	8	-0.9707	0.0435	
Brisson et al. (2020)	Canada	similarity	growth	Heracleum mantegazzianum	perennial	non-grass	field	more	8	-0.9070	0.0293	
Brisson et al. (2020)	Canada	similarity	growth	Heracleum mantegazzianum	perennial	non-grass	field	more	8	-0.0886	0.0027	
Byun & Lee (2017)	South Korea	similarity	growth	Ageratina altissima	perennial	non-grass	greenhouse	less	3	0.0190	0.0015	
Byun & Lee (2017)	South Korea	similarity	growth	Ageratina altissima	perennial	non-grass	greenhouse	less	3	-0.3006	0.0097	
Byun & Lee (2017)	South Korea	similarity	growth	Ageratina altissima	perennial	non-grass	greenhouse	less	3	-0.4308	0.0558	
Byun & Lee (2017)	South Korea	similarity	growth	Ageratina altissima	perennial	non-grass	greenhouse	less	3	-0.6931	0.1555	
Byun et al. (2013)	USA	similarity	growth	Phragmites australis	perennial	grass	outdoor	more	3	0.1759	0.0253	
Byun et al. (2013)	USA	similarity	growth	Phragmites australis	perennial	grass	outdoor	more	3	0.3759	0.1042	
Byun et al. (2013)	USA	similarity	growth	Phragmites australis	perennial	grass	outdoor	more	3	0.1603	0.2470	
Byun et al. (2013)	USA	similarity	growth	Phragmites australis	perennial	grass	outdoor	more	3	-0.1054	0.0721	
Byun et al. (2013)	USA	similarity	growth	Phragmites australis	perennial	grass	outdoor	more	3	-0.1243	0.0485	
Byun et al. (2020)	South Korea	similarity	growth	Sicyos angulatus	annual	non-grass	greenhouse	less	3	-0.8042	0.1326	
Byun et al. (2020)	South Korea	similarity	growth	Sicyos angulatus	annual	non-grass	greenhouse	less	3	-0.8487	0.0674	
Byun et al. (2020)*	South Korea	similarity	growth	Sicyos angulatus	annual	non-grass	greenhouse	less	3	-2.1285	1.7682	
Hess et al. (2022)	France	similarity	growth	Ambrosia artemisiifolia	annual	non-grass	greenhouse	between	12	0.6587	0.0190	
Hess et al. (2022)	France	similarity	growth	Ambrosia artemisiifolia	annual	non-grass	greenhouse	between	12	0.7602	0.0079	
Maron & Marler (2007)	USA	similarity	growth	Centaurea maculosa, Linaria dalmatica,  Potentilla recta	perennial	non-grass	field	more	3	-0.2332	0.2303	
Maron & Marler (2007)	USA	similarity	growth	Centaurea maculosa, Linaria dalmatica,  Potentilla recta	perennial	non-grass	field	more	3	-0.4346	0.1573	
Maron & Marler (2007)	USA	similarity	growth	Centaurea maculosa, Linaria dalmatica,  Potentilla recta	perennial	non-grass	field	more	3	-0.1967	0.2562	
Park et al. (2022)	South Korea	similarity	growth	 Festuca arundinacea	perennial	grass	greenhouse	less	3	0.0000	0.3086	
Park et al. (2022)	South Korea	similarity	growth	 Festuca arundinacea	perennial	grass	greenhouse	less	3	-0.8873	1.3200	
Park et al. (2022)	South Korea	similarity	growth	 Festuca arundinacea	perennial	grass	greenhouse	less	3	-0.4055	0.3142	
Park et al. (2022)	South Korea	similarity	growth	 Festuca arundinacea	perennial	grass	greenhouse	less	3	-0.1823	0.2636	
Quinn & Holt (2009)	USA	similarity	growth	Arundo donax	perennial	grass	field	more	6	0.1765	0.0248	
Quinn & Holt (2009)	USA	similarity	growth	Arundo donax	perennial	grass	field	more	6	0.0606	0.0277	
Sheley & James (2010)	USA	similarity	growth	Taeniatherum caput-medusae	annual	grass	field	more	4	1.8192	10.3200	
Sheley & James (2010)	USA	similarity	growth	Taeniatherum caput-medusae	annual	grass	field	more	4	0.1932	0.7009	
Sheley & James (2010)	USA	similarity	growth	Taeniatherum caput-medusae	annual	grass	field	more	4	-0.5322	0.3930	
Sheley & James (2010)	USA	similarity	growth	Taeniatherum caput-medusae	annual	grass	field	more	4	-1.0478	0.3111	
Yannelli et al. (2017a)	Germany	simi_dens	growth	Solidago gigantea	perennial	non-grass	greenhouse	less	5	1.5514	0.4112	
Yannelli et al. (2017a)*	Germany	simi_dens	growth	Solidago gigantea	perennial	non-grass	greenhouse	less	5	4.2767	72.2558	
Yannelli et al. (2017a)	Germany	simi_dens	growth	Ambrosia artemisiifolia	annual	non-grass	greenhouse	less	5	-1.5210	0.4037	
Yannelli et al. (2018)*	Germany	similarity	growth	Ambrosia artemisiifolia	annual	non-grass	greenhouse	less	6	2.9160	0.0701	
Yannelli et al. (2017b)	Germany	similarity	growth	Ambrosia artemisiifolia	annual	non-grass	greenhouse	less	6	1.7463	0.0779	
Yannelli et al. (2017b)	Germany	similarity	growth	Nassella trichotoma	annual	non-grass	greenhouse	less	6	0.2601	0.1944	
Yannelli et al. (2017b)*	Germany	similarity	growth	Solidago gigantea	perennial	non-grass	greenhouse	less	6	3.2387	0.0246	
Yannelli et al. (2017b)	Germany	similarity	growth	Solidago gigantea	perennial	non-grass	greenhouse	less	6	0.4900	0.0704	
Yannelli et al. (2017b)	Germany	similarity	growth	Solidago gigantea	perennial	non-grass	greenhouse	less	6	0.4900	0.0704	

References of studies included in the functional similarity model
Allen, P. S., & Meyer, S. E. (2014). Community structure affects annual grass weed invasion during restoration of a shrub–steppe ecosystem. Invasive Plant Science and Management, 7(1), 1-13.
Brisson, J., Teasdale, V., Boivin, P., & Lavoie, C. (2020). Plant cover restoration to inhibit seedling emergence, growth or survival of an exotic invasive plant species. Ecoscience, 27(3), 185-194. 
Byun, C., & Lee, E. J. (2017). Ecological application of biotic resistance to control the invasion of an invasive plant, Ageratina altissima. Ecology and evolution, 7(7), 2181-2192.
Byun, C., De Blois, S., & Brisson, J. (2013). Plant functional group identity and diversity determine biotic resistance to invasion by an exotic grass. Journal of Ecology, 101, 128–139 
Byun, C., Oh, M., Lee, E. J., & Kang, H. (2020). Seed density is as important as limiting similarity, diversity effect, and propagule pressure in plant restoration to control invasion. Ecological Engineering, 144, 105712.
Hess, M. C., Mesléard, F., Young, T. P., de Freitas, B., Haveneers, N., & Buisson, E. (2022). Altering native community assembly history influences the performance of an annual invader. Basic and Applied Ecology.
Maron, J. & Marler, M. (2007) Native plant diversity resists invasion at both low and high resource levels. Ecology, 88, 2651–2661
Park, S., Kim, J. H., & Lee, E. J. (2022). Resistance of plant communities to invasion by tall fescue: An experimental study combining species diversity, functional traits and nutrient levels. Basic and Applied Ecology, 58, 39-49.
Quinn, L. D., & Holt, J. S. (2009). Restoration for resistance to invasion by giant reed (Arundo donax). Invasive plant science and management, 2(4), 279-291.
Sheley, R. L., & James, J. (2010). Resistance of native plant functional groups to invasion by Medusahead (Taeniatherum caput-medusae). Invasive Plant Science and Management, 3, 294–300. 
Yannelli, F. A., Hughes, P., & Kollmann, J. (2017). Preventing plant invasions at early stages of revegetation: The role of limiting similarity in seed size and seed density. Ecological Engineering, 100, 286-290.
Yannelli, F. A., Karrer, G., Hall, R., Kollmann, J., & Heger, T. (2018). Seed density is more effective than multitrait limiting similarity in controlling grassland resistance against plant invasions in mesocosms. Applied Vegetation Science, 21(3), 411-418.
Yannelli, F. A., Koch, C., Jeschke, J. M., and Kollmann, J. (2017b). Limiting similarity and Darwin's naturalization hypothesis: understanding the drivers of biotic resistance against invasive plant species. Oecologia 183, 775–784. doi: 10.1007/s00442-016-3798-8


Supplementary Data 1c. Basic data of the selected publications (n=7) and resulting data points (k=43) of the seeding density model. The seeding density model considered low density seeding of native species as control and high-density seeding of native species as treatment. Effect size used was log response ration (lnRR). Studies involved into the model are listed below. * Excluded as outliers.
Source	Country	Studied mechanism	Performance indicator	Invasive alien species	Life form	Functional group	Experiment type	Experiment lenght	Number of samples	lnRR	var (lnRR)	
Byun et al. (2020)	South Korea	density	growth	Sicyos angulatus	annual	non-grass	greenhouse	less	3	-0.5306	1.7203	
Byun et al. (2020)	South Korea	density	growth	Sicyos angulatus	annual	non-grass	greenhouse	less	3	-1.0415	0.9747	
Firn et al. (2010)	Australia	density	growth	Eragrostis curvula	perennial	grass	greenhouse	between	3	0.1603	0.3600	
Firn et al. (2010)	Australia	density	growth	Eragrostis curvula	perennial	grass	greenhouse	between	3	-0.9555	1.1378	
Firn et al. (2010)	Australia	density	growth	Eragrostis curvula	perennial	grass	greenhouse	between	3	0.1652	0.0060	
Firn et al. (2010)	Australia	density	growth	Eragrostis curvula	perennial	grass	greenhouse	between	3	0.0457	0.0022	
Schantz et al. (2015)	USA	density	growth	Bromus tectorum	annual	grass	field	more	3	0.1030	0.0802	
Schantz et al. (2015)	USA	density	growth	Bromus tectorum	annual	grass	field	more	3	0.3766	0.0662	
Schantz et al. (2015)	USA	density	growth	Bromus tectorum	annual	grass	field	more	3	0.4335	0.0669	
Schantz et al. (2019)	USA	density	establishment	Taniatherum caput-medusae, Bromus tectorum	annual	grass	field	more	3	-0.0290	0.0012	
Schantz et al. (2019)	USA	density	establishment	Taniatherum caput-medusae, Bromus tectorum	annual	grass	field	more	3	0.0910	0.0011	
Schantz et al. (2019)	USA	density	establishment	Taniatherum caput-medusae, Bromus tectorum	annual	grass	field	more	3	-0.0588	0.0012	
Tarsa et al. (2022)	USA	density	growth	Phalaris arundinacea 	perennial	grass	outdoor	less	6	-0.3782	0.1294	
Tarsa et al. (2022)	USA	density	growth	Phalaris arundinacea 	perennial	grass	outdoor	less	6	-0.4415	0.1397	
Tarsa et al. (2022)*	USA	density	growth	Phalaris arundinacea 	perennial	grass	outdoor	less	6	-4.3213	89.9935	
Yannelli et al. (2017)	Germany	density	growth	Solidago gigantea	perennial	non-grass	greenhouse	less	5	0.2451	0.0061	
Yannelli et al. (2017)*	Germany	density	growth	Solidago gigantea	perennial	non-grass	greenhouse	less	5	2.9704	72.6609	
Yannelli et al. (2017)	Germany	density	growth	Ambrosia artemisiifolia	annual	non-grass	greenhouse	less	5	0.3785	0.0132	
Yu et al. (2020)	China	density	growth	Mikania micrantha	perennial	non-grass	outdoor	less	15	-0.5206	0.0055	
Yu et al. (2020)	China	density	growth	Mikania micrantha	perennial	non-grass	outdoor	less	15	-0.8130	0.0036	
Yu et al. (2020)	China	density	growth	Mikania micrantha	perennial	non-grass	outdoor	less	15	-1.4691	0.0066	

References of studies included in seeding density model
Byun, C., Oh, M., Lee, E. J., & Kang, H. (2020). Seed density is as important as limiting similarity, diversity effect, and propagule pressure in plant restoration to control invasion. Ecological Engineering, 144, 105712.
Firn, J., MacDougall, A. S., Schmidt, S., & Buckley, Y. M. (2010). Early emergence and resource availability can competitively favour natives over a functionally similar invader. Oecologia, 163(3), 775-784.
Schantz, M. C., Sheley, R. L., & James, J. J. (2015). Role of propagule pressure and priority effects on seedlings during invasion and restoration of shrub-steppe. Biological Invasions, 17(1), 73-85.
Schantz, M. C., Sheley, R. L., & James, J. J. (2019). Propagule pressure and priority seeding effects on the demography of invasive annual and native perennial grass species. Plant Ecology & Diversity, 12(2), 139-150.
Tarsa, E. E., Holdaway, B. M., & Kettenring, K. M. (2022). Tipping the balance: The role of seed density, abiotic filters, and priority effects in seed?based wetland restoration. Ecological Applications, 32(8), e2706.
Yannelli, F. A., Hughes, P., & Kollmann, J. (2017). Preventing plant invasions at early stages of revegetation: The role of limiting similarity in seed size and seed density. Ecological Engineering, 100, 286-290.
Yu, H., Yue, M., Wang, C., Le Roux, J. J., Peng, C., & Li, W. (2020). Priority effects and competition by a native species inhibit an invasive species and may assist restoration. Ecology and Evolution, 10(23), 13355-13369.


Supplementary Data 1d. Basic data of the selected publications (n=11) and resulting data points (k=31) of the priority effect model. The effect model considered low priority seeding of native species as control and high priority seeding of native species as treatment. Effect size used was log response ration (lnRR). Studies involved into the model are listed below. * Excluded as outliers.
Source	Country	Studied mechanism	Performance indicator	Invasive alien species	Life form	Functional group	Experiment type	Experiment length	Number of samples	l RR	var (lnRR)	
Cleland et al. (2015)	USA	priority	growth	Bromus hordeaceus	annual	grass	outdoor	less	8	-0.1226	0.0242	
Cleland et al. (2015)	USA	priority	growth	Trifolium hirtum	annual	non-grass	outdoor	less	8	0.2255	0.1089	
Cleland et al. (2015)	USA	priority	growth	Trifolium hirtum	annual	non-grass	outdoor	less	8	0.1112	0.0864	
Cleland et al. (2015)	USA	priority	growth	Bromus hordeaceus	annual	grass	outdoor	less	8	0.5067	0.0643	
Cleland et al. (2015)	USA	priority	growth	Bromus hordeaceus	annual	grass	outdoor	less	8	0.3827	0.0294	
Cleland et al. (2015)	USA	priority	growth	Bromus hordeaceus	annual	grass	outdoor	less	8	-0.6995	0.0985	
Cleland et al. (2015)	USA	priority	growth	Lolium multiflorum	perennial	grass	outdoor	less	8	-0.6423	0.0184	
Cleland et al. (2015)	USA	priority	growth	Lolium multiflorum	perennial	grass	outdoor	less	8	0.5733	0.0557	
Cleland et al. (2015)	USA	priority	growth	Lolium multiflorum	perennial	grass	outdoor	less	8	0.6931	0.0920	
Cleland et al. (2015)	USA	priority	growth	Lolium multiflorum	perennial	grass	outdoor	less	8	0.2451	0.0423	
Delory et al. (2019)*	Germany	priority	growth	Senecio inaequidens	perennial	non-grass	greenhouse	less	5	-3.6889	0.1597	
Delory et al. (2019)	Germany	priority	growth	Senecio inaequidens	perennial	non-grass	greenhouse	less	5	-2.9755	0.6637	
Grman & Suding (2010)	USA	priority	growth	more species	annual	NA	outdoor	less	7	-1.7664	0.1707	
Hess et al. (2020)	France	priority	growth	Cortaderia selloana	annual	non-grass	greenhouse	between	5	-0.0290	0.0000	
Hess et al. (2020)	France	priority	establishment	Ambrosia artemisiifolia	annual	non-grass	greenhouse	between	5	-0.0740	0.0002	
Hess et al. (2020)	France	priority	establishment	Bothriochloa barbinodis	annual	non-grass	greenhouse	between	5	-0.0074	0.0005	
Hess et al. (2020)	France	priority	establishment	Cortaderia selloana	annual	non-grass	greenhouse	between	5	-0.0443	0.0002	
Lang et al. (2017)	Germany	priority	growth	Cytisus scoparius	perennial	non-grass	greenhouse	between	15	0.1349	0.0013	
Perkins & Hatfield (2014)	USA	priority	growth	Agropyron cristatum	perennial	grass	greenhouse	less	9	-0.8622	0.0522	
Perkins & Hatfield (2014)	USA	priority	growth	Bromus tectorum	annual	grass	greenhouse	less	9	-0.5831	0.0168	
Perkins & Hatfield (2014)	USA	priority	growth	Taeniatherum caput-medusae 	annual	grass	greenhouse	less	9	-0.5965	0.0302	
Stevens & Fehmi (2011)	USA	priority	growth	Pennisetum ciliare	perennial	grass	greenhouse	less	5	-1.5950	0.0705	
Stevens & Fehmi (2011)	USA	priority	growth	Pennisetum ciliare	perennial	grass	greenhouse	less	5	-0.9163	18.0159	
Tarsa et al. (2022)	USA	priority	growth	Phalaris arundinacea 	perennial	grass	outdoor	less	6	-0.3610	0.0750	
Tarsa et al. (2022)	USA	priority	growth	Phalaris arundinacea 	perennial	grass	outdoor	less	6	-3.0910	0.0278	
Ulrich & Perkins (2014)	USA	priority	growth	Bromus inermis	perennial	grass	greenhouse	less	10	-2.3775	0.1179	
Ulrich & Perkins (2014)	USA	priority	growth	Poa pratensis	perennial	grass	greenhouse	less	10	-1.1206	0.1685	
Wohlwend et al. (2019)	USA	priority	growth	Lespedeza cuneata	perennial	non-grass	field	more	51	-1.2937	0.0139	
Young et al. (2014)	USA	priority	growth	Vulpia (Festuca) myuros/V. bromoides, Bromus hordeaceus, Hordeum murinum, Avena barbata/A. fatua	annual	grass	field	between	5	-1.4508	0.2555	
Young et al. (2014)	USA	priority	growth	Vulpia (Festuca) myuros/V. bromoides, Bromus hordeaceus, Hordeum murinum, Avena barbata/A. fatua	annual	grass	field	between	5	-0.4246	0.0485	
Young et al. (2014)	USA	priority	growth	Vulpia (Festuca) myuros/V. bromoides, Bromus hordeaceus, Hordeum murinum, Avena barbata/A. fatua	annual	grass	field	between	5	-0.2288	0.1154	

References of studies included in priority effect model
Cleland, E. E., Esch, E., & McKinney, J. (2015). Priority effects vary with species identity and origin in an experiment varying the timing of seed arrival. Oikos, 124(1), 33-40.
Delory, B.M., Weidlich, E.W., Kunz, M., Neitzel, J. and Temperton, V.M. (2019) The exotic species Senecio inaequidens pays the price for arriving late in temperate European grassland communities. Oecologia, 191, 657-671. http://doi.org/10.1007/s00442-019-04521-x
GrmanN E & Suding KN (2010) Within-year soil legacies contribute to strong priority effects of exotics on native California grassland communities. Restoration Ecology 18, 664–670.
Hess, M. C. M., Buisson, E., Fontes, H., Bacon, L., Sabatier, F., & Mesléard, F. (2020). Giving recipient communities a greater head start and including productive species boosts early resistance to invasion. Applied Vegetation Science, 23(3), 340-352.
Lang, M., Hanslin, H. M., Kollmann, J., & Wagner, T. (2017). Suppression of an invasive legume by a native grass—High impact of priority effects. Basic and applied ecology, 22, 20-27.
Perkins, L. B., & Hatfield, G. (2014). Competition, legacy, and priority and the success of three invasive species. Biological invasions, 16(12), 2543-2550.
Stevens, J. M., and J. S. Fehmi. 2011. Early establishment of a native grass reduces the competitive effect of a nonnative grass. Restoration Ecology 19:399–406
Tarsa, E. E., Holdaway, B. M., & Kettenring, K. M. (2022). Tipping the balance: The role of seed density, abiotic filters, and priority effects in seed?based wetland restoration. Ecological Applications, 32(8), e2706.
Ulrich E, Perkins L (2014) Bromus inermis and Elymus canadensis but not Poa pratensis demonstrate strong competitive effects and all benefit from priority. Plant Ecology 215:1269–1275
Wohlwend, M. R., Schutzenhofer, M. R., & Knight, T. M. (2019). Long?term experiment manipulating community assembly results in favorable restoration outcomes for invaded prairies. Restoration Ecology, 27(6), 1307-1316.
Young, T. P., Zefferman, E. P., Vaughn, K. J., & Fick, S. (2014). Initial success of native grasses is contingent on multiple interactions among exotic grass competition, temporal priority, rainfall and site effects. AoB Plants, 7, plu081
