# Supplementary material for: Meta-analysis identifies native priority as a mechanism that supports the restoration of invasion-resistant plant communities
Source: Commun Biol. 2023 Oct 30;6:1100. doi: 10.1038/s42003-023-05485-8 (PMC10616274; doi:10.1038/s42003-023-05485-8)
Supplement: Supplementary file 5 — Reporting Summary [file 42003_2023_5485_MOESM5_ESM.pdf]

nature portfolio

Corresponding author(s): Melinda Halassy
Last updated by author(s): Oct 3, 2023

Reporting Summary

Nature Portfolio wishes to improve the reproducibility of the work that we publish. This form provides structure for consistency and transparency in reporting. For further information on Nature Portfolio policies, see our Editorial Policies and the Editorial Policy Checklist.

Statistics

For all statistical analyses, confirm that the following items are present in the figure legend, table legend, main text, or Methods section.

n/a	Confirmed	
☐	☒	The exact sample size (*n*) for each experimental group/condition, given as a discrete number and unit of measurement
☐	☒	A statement on whether measurements were taken from distinct samples or whether the same sample was measured repeatedly
☐	☒	The statistical test(s) used AND whether they are one- or two-sided<br>*Only common tests should be described solely by name; describe more complex techniques in the Methods section.*
☐	☒	A description of all covariates tested
☐	☒	A description of any assumptions or corrections, such as tests of normality and adjustment for multiple comparisons
☐	☒	A full description of the statistical parameters including central tendency (e.g. means) or other basic estimates (e.g. regression coefficient) AND variation (e.g. standard deviation) or associated estimates of uncertainty (e.g. confidence intervals)
☐	☒	For null hypothesis testing, the test statistic (e.g. *F*, *t*, *r*) with confidence intervals, effect sizes, degrees of freedom and *P* value noted<br>*Give P values as exact values whenever suitable.*
☐	☒	For Bayesian analysis, information on the choice of priors and Markov chain Monte Carlo settings
☐	☒	For hierarchical and complex designs, identification of the appropriate level for tests and full reporting of outcomes
☐	☒	Estimates of effect sizes (e.g. Cohen's *d*, Pearson's *r*), indicating how they were calculated

Our web collection on statistics for biologists contains articles on many of the points above.

Software and code

Policy information about availability of computer code

Data collection	ISI Web of Science database (Science Citation Index Expanded edition) + R programming environment Version 3.4.4.76, metaDigitise package
Data analysis	R programming environment Version 3.4.4.76, R-packages metafor and multicomp and orchaRd 2.0

For manuscripts utilizing custom algorithms or software that are central to the research but not yet described in published literature, software must be made available to editors and reviewers. We strongly encourage code deposition in a community repository (e.g. GitHub). See the Nature Portfolio guidelines for submitting code & software for further information.

Data

Policy information about availability of data

All manuscripts must include a data availability statement. This statement should provide the following information, where applicable:

- Accession codes, unique identifiers, or web links for publicly available datasets
- A description of any restrictions on data availability
- For clinical datasets or third party data, please ensure that the statement adheres to our policy

The authors declare that the data supporting the findings of this study are available within the article, and its Supplementary information files. Any other relevant data are available from the corresponding author upon reasonable request.

Research involving human participants, their data, or biological material

Policy information about studies with human participants or human data. See also policy information about sex, gender (identity/presentation), and sexual orientation and race, ethnicity and racism.

Reporting on sex and gender	do not apply
Reporting on race, ethnicity, or other socially relevant groupings	do not apply
Population characteristics	do not apply
Recruitment	do not apply
Ethics oversight	do not apply

Note that full information on the approval of the study protocol must also be provided in the manuscript.

Field-specific reporting

Please select the one below that is the best fit for your research. If you are not sure, read the appropriate sections before making your selection.

☐ Life sciences ☐ Behavioural & social sciences ☒ Ecological, evolutionary & environmental sciences

For a reference copy of the document with all sections, see nature.com/documents/nr-reporting-summary-flat.pdf

Ecological, evolutionary & environmental sciences study design

All studies must disclose on these points even when the disclosure is negative.

Study description

We performed four models in a meta-analysis in the frame of a systematic review to provide an overview of current trends and future prospects for increasing biological resistance to invasive alien plant species in ecological restoration based on functional similarity, seeding density and native species priority.

Research sample

Our PICO table is in the Supplementary Materials:
Population: Experimental restoration studies conducted in terrestrial plant communities
Intervention: Active introduction of native species and IAS via seeding or planting with manipulation of (a) functional similarity, seeding density and priority effect (focal study), (b) functional similarity, (c) seeding density, and (d) priority effect
Comparators:
(a) control: only IAS, treatment: IAS + high similarity, high density, high priority native species
(b) control: IAS + low similarity native species, treatment: IAS + high similarity native species
(c) control: IAS + low density native species, treatment: IAS + high density native species
(d) control: concurrent seeding of IAS + native species, treatment: native priority + IAS
Outcome: Establishment of IAS (e.g. seedling emergence) or Growth of IAS (e.g. percentage cover, biomass)

Sampling strategy

We systematically screened the literature for restoration experimental studies including seeding or planting on biotic resistance of native species or communities towards invasive alien species due to functional similarity, seeding density, and native priority.
The search was performed using the ISI Web of Science database (Science Citation Index Expanded edition) on 16 March 2022 and then updated on 7 February 2023. We used the exact search feature with the search strings for invasion, active introduction of species and the three studied mechanisms of resistance: ALL=((invasi*) AND (seeding OR sow* OR planting) AND ("functional similarity" OR "plant trait" OR "seed density" OR "seeding rate" OR "propagule pressure" OR "priority" OR "arrival order")). We retained only publications in English with no limit for publication date. The search yielded 202 records.
We only retained articles that met the following criteria (see also our PICO model in Supplementary Table 1): (1) focused on restoration-oriented experimental studies conducted in terrestrial plant communities; (2) included active introduction of native species and invasive alien species via seeding or planting; (3) involved at least one native species and one invasive alien species; (4) and at least one of the following aspects:

- functional similarity: there were at least two different native species involved, which differ in functional similarity to the invasive alien species;
- seeding density: the native species were sown at a minimum of two different seed densities;
- priority effect: native species have been sown on at least two occasions, at the same time as and before invasive alien species.

The initial title screening that focused on experimental studies in terrestrial vegetation only reduced the list further down to 121. These items were then checked for the abstract, leaving 54 records where the full text was downloaded for a thorough analysis to determine whether they provided any answers to our research questions. This reading resulted in 31 papers. We additionally searched for relevant references in recent reviews on the topic and in the selected papers in our research. The search yielded additional 17 records. The PRISMA flow chart shows the whole screening process (Supplementary Figure 1)

Data collection

Mean, replication (N) and standard deviation estimates (s.d., s.e.m. or 95% CI) for both control and treatment were compiled for the performance of invasive alien species. Data were extracted from text or tables or read from figures using the metaDigitise R package.

Data from the same publication, but for different species or mechanisms were collected as separate data points. For multi-year studies, only data from the last year were extracted. If multiple indicators were included in a publication, biomass was preferred for abundance and seedling survival for establishment. The logarithmic response rate (lnRR) was calculated as an estimate of the effect size, as it is not affected by different variances between control and treated groups, and the results are easy to interpret. The replication number was not available for one publication, which was excluded from our analysis. In some papers, relative competition intensity (RCI = (Pcontr − Ptreat)/Ptreat) was reported that we converted to lnRR using the following equitation: lnRR = ln(Pcontr/Ptreat) = −ln(1 − RCI). Additionally, from each selected publication, we collected the following information: the publication year, the country, the study type (field or greenhouse experiment), the native species and invasive alien species involved, and the mechanisms studied. Special focus was on the details of the studied three basic mechanisms related to biotic resistance, such as the functional groups or plant traits considered, seeding densities used for native species and invasive alien species, and the difference between seeding/planting times of introduced plants used in priority studies. We also collected information on the types of habitats involved, the plant form and number of individuals applied, the temporal and spatial scale of the experiments, the details of treatments and maintenance and the performance indicators used. Web of Science search and data extraction with MetaDigitise R package was done by Melinda Halassy, All other search and data extraction included OV, KT and ACs

Timing and spatial scale	The search was performed using the ISI Web of Science database (Science Citation Index Expanded edition) on 16 March 2022 and then updated on 7 February 2023. We retained only publications in English with no limit for publication date.
Data exclusions	We only retained articles that met the following criteria (see also our PICO model in Supplementary Table 1): (1) focused on restoration-oriented experimental studies conducted in terrestrial plant communities; (2) included active introduction of native species and invasive alien species via seeding or planting; (3) involved at least one native species and one invasive alien species; (4) and at least one of the following aspects:<br>● functional similarity: there were at least two different native species involved, which differ in functional similarity to the invasive alien species;<br>● seeding density: the native species were sown at a minimum of two different seed densities;<br>● priority effect: native species have been sown on at least two occasions, at the same time as and before invasive alien species.<br><br>For multi-year studies, only data from the last year were extracted. If multiple indicators were included in a publication, biomass was preferred for abundance and seedling survival for establishment. We carried out sensitivity analyses for each model by checking results with and without including outliers defined using graphical methods (boxplots), and removed the outliers and data points with extreme high variability of lnRR to achieve better model performance based on Akaike's information criterion (Supplement Table 8).
Reproducibility	We give detailed description of the systematic review process and provide final lists of publications and the extracted lnRR and var (lnRR) data and examples of R codes in the Supplementary Notes.
Randomization	Randomization do not apply. Study ID was treated as a random effect in all models to account for the non-independence of individual effect sizes calculated from the same study.
Blinding	Blinding do not apply, we did not perform direct studies, but extracted data for published literature.

Did the study involve field work? ☐ Yes ☒ No

Reporting for specific materials, systems and methods

We require information from authors about some types of materials, experimental systems and methods used in many studies. Here, indicate whether each material, system or method listed is relevant to your study. If you are not sure if a list item applies to your research, read the appropriate section before selecting a response.

Materials & experimental systems

n/a	Involved in the study
☒	☐ Antibodies
☒	☐ Eukaryotic cell lines
☒	☐ Palaeontology and archaeology
☒	☐ Animals and other organisms
☒	☐ Clinical data
☒	☐ Dual use research of concern
☒	☐ Plants

Methods

n/a	Involved in the study
☒	☐ ChIP-seq
☒	☐ Flow cytometry
☒	☐ MRI-based neuroimaging
